# Selective enrichment on a wide polysaccharide spectrum allowed isolation of novel metabolic and taxonomic groups of haloarchaea from hypersaline lakes

**DOI:** 10.3389/fmicb.2022.1059347

**Published:** 2022-11-23

**Authors:** Dimitry Y. Sorokin, Alexander G. Elcheninov, Tatiana V. Khijniak, Tatiana V. Kolganova, Ilya V. Kublanov

**Affiliations:** ^1^Winogradsky Institute of Microbiology, Federal Research Centre of Biotechnology, Russian Academy of Sciences, Moscow, Russia; ^2^Department of Biotechnology, Delft University of Technology, Delft, Netherlands; ^3^Institute of Bioengineering, Federal Research Centre of Biotechnology, Russian Academy of Sciences, Moscow, Russia; ^4^Faculty of Biology, Lomonosov Moscow State University, Moscow, Russia

**Keywords:** halo(natrono)archaea, hypersaline lakes, soda lakes, polysaccharides, hydrolytic

## Abstract

Extremely halophilic archaea (haloarchaea) of the class *Halobacteria* is a dominant group of aerobic heterotrophic prokaryotic communities in salt-saturated habitats, such as salt lakes and solar salterns. Most of the pure cultures of haloarchaea were enriched, isolated, and cultivated on rich soluble substrates such as amino acids, peptides or simple sugars. So far, the evidences on the capability of haloarchaea to use different polysaccharides as growth substrates remained scarce. However, it is becoming increasingly obvious that these archaea can also actively participate in mineralization of complex biopolymers, in particular cellulose and chitin–two dominant biomass polysaccharides on the planet. Here we used an array of commercially available homo- and heteropolysaccharides to enrich hydrolytic haloarchaea from hypersaline salt lakes with neutral pH and from alkaline soda lakes. This resulted in isolation of a range of halo- and natrono-archaea, respectively, belonging to already described taxa as well as several new genus-level lineages. In some cases, the isolates enriched with different polysaccharides happened to be closely related, thus representing generalistic ecotype, while the others were narrow specialists. In general, soda lakes yielded a broader range of polysaccharide-utilizing specialists in comparison to neutral salt lakes. The results demonstrated a significant diversity of halo(natrono)archaea with a previously unrecognized potential for utilization of a broad range of natural polysaccharides in hypersaline habitats.

## Introduction

Hypersaline lakes and solar salterns at its final evaporation stage represent unique salt-saturated habitats dominated by extremely halophilic microbial communities among which the extremely halophilic archaea of the class *Halobacteria* is the particularly successful group (Cui and Dyall-Smith, 2021). These archaea (at least those known in culture) are mostly aerobic organoheterotrophs, utilizing simple soluble organic compounds, such as amino acids and sugars (Andrei et al., 2012; Oren, 2013, 2015; Grant and Jones, 2016). Haloarchaea typically have very high cell density that gives the characteristic reddish color to hypersaline brines in intracontinental athalassic lakes and thalassic endeavaporite pools of the marine solar salt concentrators. Only handful of cultivated haloarchaeal species can grow with polymeric substances, such as starch, proteins or olive oil (Bhatnagar et al., 2005; Enache and Kamekura, 2010; Moshfegh et al., 2013; Selim et al., 2014; Amoozegar et al., 2017). Recently, this spectrum has been expanded by recalcitrant insoluble polysaccharides, such as cellulose and chitin as well as some other partially soluble polysaccharides, such as galactomannan and xylan. Utilization of native insoluble forms of cellulose has recently been shown for the neutrophilic genera *Halococcoides*, *Halomicrobium*, and *Halosimplex* (Sorokin et al., 2015, 2019a,2020a) and for two genera of natronoarchaea from soda lakes–*Natronolimnobius* and *Natronobiforma* (Sorokin et al., 2015, 2018, 2019b). The ability to use chitin as the growth substrate has been proven for the neutrophilic genera *Halomicrobium* and *Salinarchaeum* and for the natronoarchael genus *Natrarchaeobius* (Sorokin et al., 2015, 2019c,2020b; Minegishi et al., 2017). Finally, growth with locust bean galacto-beta-1,4-mannan was shown for the neutrophilic genera *Natronoarchaeum* and *Haloarcula* (Shimane et al., 2010; Enomoto et al., 2020).

The potential of haloarchaea to utilize various recalcitrant polysaccharides produced mostly by plants and algae is of significant interest both for fundamental understanding of their functional importance for the organic matter mineralization in hypersaline environments and also by regarding them as a source of extremely halo(alkali)stable extracellular hydrolases which have important application potential in production of biofuel from lignocellulosic wastes because this process often starts with a decrystallization pretreatment step, performed either with alkali or ionic liquids (Kaar and Holtzapple, 2000; Zavrel et al., 2010; Begemann et al., 2011).

In this work, the search for polysaccharide-utilizing haloarchaea was extended beyond the most abundant cellulose and chitin. For this, a range of commercially available polysaccharides of plant and microbial origin was used for selective enrichment and further isolation in pure cultures of halo(natrono)archaea able to utilize these polymers as growth substrate. The *de novo* sequenced genomes of these strains allowed to establish their phylogenies as well as to detect the genes, encoding enzymes responsible for their polysacharidolytic capacities. The results demonstrated significant diversity of polysaccharide-specialized haloarchaea belonging to already described genera and species (mostly for salt lakes) and several new genera (mostly among natronoarchaea), all of which have enzymatic repertoire sufficient for decomposition of the respective polysaccharides.

## Experimental procedures

### Samples

Sediment (top 3 cm) and brine samples were obtained from five hypersaline chloride-sulfate lakes with neutral pH in Kulunda Steppe (Altai, Russia) and from hypersaline alkaline (soda) lakes in Kulunda Steppe (three lakes), northeastern Mongolia (two lakes) and North America (California, two lakes) (Sorokin et al., 2015). Two “master mixes,” one for each type of lakes, were created by mixing equal parts of sediments and brines from each lake and used at 5% (v/v) for primary enrichments.

### Enrichment and growth conditions

The neutrophilic haloarchaea originated from salt lakes were enriched, purified and further cultivated in a neutral base medium 1 with the following composition (g l^–1^): 230 NaCl, 5 KCl, 0.2 NH_4_Cl, 2.5 K_2_HPO_4_, pH 6.8. After sterilization, the base was supplemented with vitamin and trace metal mix (Pfennig and Lippert, 1966) (1 ml l^–1^ each) and 2 mM MgSO_4_. For the soda lake enrichments and further cultivation of alkaliphilic natronoarchaea, a sodium carbonate/bicarbonate-based medium 2 containing 4 M total Na^+^ [(g l^–1^): 190 Na_2_CO_3_, 30 NaHCO_3_, 16 NaCl, 5 KCl and 1 K_2_HPO_4_, final pH 10 after sterilization] was supplemented with the same additions as for the medium 1, except that the amount of Mg was two times lower and that 4 mM NH_4_Cl was added after sterilization. This alkaline medium was mixed 1:3 with the neutral medium 1, resulting in the final pH of 9.6.

Polysaccharides (Sigma-Aldrich and Megazyme; Supplementary Table 1) were either added from suspensions in sterile distilled water (when heat sterilization was not possible) or from 5% heat-sterilized (110°C for 20 min) stocks to a final concentration of 0.5 g l^–1^. At the stage of initial enrichments and further 1:100 transfers, a mixture of streptomycin and kanamycin (final concentration 100 mg l^–1^) was added to suppress bacterial development. Cultivation was performed in 30 ml bottles sealed with gray-rubber septa (to prevent evaporation) containing 10 ml medium at 35°C on a rotary shaker at 150 rpm. Solid media were prepared by 3:2 (v:v) mixing of the fully prepared liquid media with 4% washed agar at 50°C. Solid NaCl was added to the portions of liquid media before heating to bring the salinity back to 4 M of total Na^+^ after agar addition. For isolation of pure cultures, the initial positive enrichments were passed 2 times into new media containing target polysaccharides at 1:100 dilution to obtain sediment-free cultures, followed with dilution to extinction series and finally plating the maximal positive dilutions onto solid media with the same composition. The dominant colony types or those showed visible signs of polymer degradation (where possible) were picked up with sterile Pasteur capillary with pooled tips under control of binocular and placed into liquid media. Only those cultures which showed vigorous growth in liquid media were further purified by repeating the colony formation procedure. The purity of isolates were confirmed by microscopy, 16S-rRNA gene sequencing (Sanger and amplicon profiling) and in several cases by full genome sequencing.

### Polysaccharide utilization activity

The main indication of polysaccharide utilization was consistent microbial growth in liquid culture whereby the polysaccharide in question served as the sole carbon and energy source. In addition, whenever it was possible, the hydrolytic activities of spot-colonies were also visualized on agar plates, either by formation of clearance zones (amorphous cellulose, beta-mannan) or reagent-developed hydrolysis zone: Lugol solution for starch, pullulan and pectin and Congo Red/1 M NaCl for xylan, xyloglucan, arabonoxylan, and glucomannan.

### Genomic sequencing and phylogenetic analysis

Genomic DNA isolation, DNA library preparation, sequencing as well as genome assembly were performed as described earlier (Sorokin et al., 2022a).

Phylogenomic analysis based on the “ar122” set of conserved single copy archaeal proteins (Rinke et al., 2021) was performed as follows: the protein sequences were identified and aligned in *in silico* proteomes of the type species of all genera within *Halobacteria* class (non-type species were taken for *Halalkalicoccus* and *Natronoarchaeum* genera because the genomes of the type species were not available) using the GTDB-tk v.1.7.0 with reference data v.202 (Chaumeil et al., 2019). The maximum likelihood tree was inferred using the RAxML v.8.2.12 (Stamatakis, 2014) with the PROTGAMMAILG model of amino acid substitution; support values were calculated from the 1000 rapid bootstrap replications. The phylogenetic tree was polished using iTOL v.6.5.8 (Letunic and Bork, 2019).

### Genome analysis

The genomes were annotated with NCBI Prokaryotic Genome Annotation Pipeline (Tatusova et al., 2016). Carbohydrate-active enzymes (CAZymes) including glycosidases, polysaccharide lyases, carbohydrate esterases, glycosyl transferases, and carbohydrate oxidases genes were predicted using dbCAN2 script v2.0.11 (Zhang et al., 2018) with HMMER v3.3 (Mistry et al., 2013) with default thresholds. The most probable activities of identified CAZymes (excluding glycosyl transferases, for which the manual verification of the predictions was not performed) were predicted using BLAST search against Swiss-Prot database (Boutet et al., 2016).

### Genbank accession numbers

The 16S rRNA gene sequences generated in this study were deposited in the GenBank under accession numbers ON787970-ON788000. The whole genome sequence are available in GenBank under the following accession numbers: JAOPJY000000000, JAOPJZ000000000, JAOPKA000000000, JAOPKB000000000, JAOPKC000000000, JAOPKD000000000, and JAOPKE000000000.

## Results

### Polysaccharide-utilizing haloarchaea from hypersaline lakes with neutral pH

Positive enrichment cultures from salt lakes were obtained with 13 out of 18 polysaccharide compounds tested. The fastest development (maximal growth yield was achieved after 1 week) was observed with starch-like compounds (amylopectin, pullulan, and glycogen), while the slowest (up to a month)–with insoluble beta-linked polysaccharides (beta-mannan and curdlan). Other positive cultures showed growth in between 2 and 3 weeks. All positive primary enrichments were transferred into a sediment-free stage after 2–3 consecutive 1:100 (v:v) transfers on the same synthetic medium and acquired pink coloration with a domination of polymorphic flat cells characteristic of haloarchaea accompanied by visible degradation of substrates in case of insoluble polysaccharides. Further dilutions to extinction were performed without antibiotics and were generally positive up to 10^–8^–10^–9^. Final purification was achieved by isolation of individual colonies on solid media. Pure cultures of polysacharidolytic haloarchaea were obtained from those colony morphotype(s) (not always dominant ones) which consistently grew back in liquid medium with the target polysaccharide used in the enrichment. The list of isolates is given in Table 1.

**TABLE 1:** Polysaccharide-utilizing neutrophilic haloarchaea enriched and isolated from hypersaline salt lakes with neutral pH.

Polysaccharide	Isolates	Identification by 16S rRNA gene sequence		Spectrum of utilized polysaccharides	
		Closest cultured relative	% identity	Stable growth in liquid culture	Colony activity (zone of hydrolysis, mm/2–3 weeks)
**Alpha-glucans**					
Amylopectin	HArc-St	*Natrinema salaciae*	99	Amp/Sst/Glc/Arx	nd/41/nd
Pullulan	HArc-pul1	*Haloferax alexandrinus*	100	Pul/Sst/Arx	30/20/nd
	HArc-pul2	*Natronoarchaeum rubrum*	99	Pul/Sst/Arx/Gtm/Glt/Crd	45/50/nd
Glycogen	HArc-glc1	*Halorubrum alkalophilum*	99	Glc/Sst	nd/22
	HArc-glc2	*“Halobacterium hubeiense”*	99	Glc/Sst/Arx	nd/15
Arabinan	HArc-arb1-5	*“Halobacterium hubeiense”*	99	Arb/Arx/Xyl	nd/40/20
Dextran	No isolates; not utilized by any isolates				
**Beta-fructans**					
Levan	HArc-lev	*Halorhabdus tiamatea*	99	Lev/Sst/Arx/Pul/Xyl	nd/23
Inulin	No isolates; not utilized by any isolates				
**Beta-bonded polysaccharides**					
Pectic galactan	HArc-glct1	*Natronoarchaeum rubrum*	99	Glt/Pul/Sst/Arx/Pul/Xyl	20/30/10/20/20/30
Beta-mannan	HArc-m1*	*Halovarius/Haloterrigena*	**94–95**	Man/Gcm/Arx/Xyl/Xgl/Ac	20/20/30/30/nd/32/15
Glucomannan	HArc-gm1/3/4	*Halorhabdus tiamatea*	99	Pul/Sst/Arx/Xyl	30/40/30/30
	HArc-gm2	*Halomicrobium zhouii*	99	Gcm/Man/Arx/Xyl/Ac/Lam/Bgl	20/8/40/30/7/nd/nd
Galactomannan	HArc-glctm2	*Haloarcula hispanica*	97.3	Gtm/Glt/Pul/Sst	nd/nd/18/12
	HArc-glctm4	*Natronoarchaeum rubrum*	99		
Xylan	HArc-x1/2	*Halorubrum tebenquichense*	100	Xyl/Arx	30/22
Xyloglucan	HArc-xlg1	*Halorhabdus tiamatea*	99.8	Xgl/Arx/Xyl	nd/25/10
Arabinoxylan	HArc-ax1/3	*Halorhabdus tiamatea*	99.7	Pul/Sst/Arx/Xyl	27/34/25/32
Curdlan	HArc-curdl1	*Haloferax sulfurifontis*	97.6	Crd/Pch/Lam/Pul(w)	nd
	HArc-curdl4	*Natronoarchaeum rubrum*	99	Crd/Pch/Lam/Pul(w)	nd/nd/nd/16
	**HArc-curdl5-1**	*Halapricum salinarum*	**95**	Crd/Pch/Lam/Sst(w)/Inl/Glc	nd
Arabinogalactan	No isolates; not utilized by any isolates				
Alginate					
Pectin					

Polysaccharides: Sst, soluble starch; Glc, glycogen; Pul, pullulan; Lev, levan; Inl, inulin; Amp, amylopectin; Arb, arabinan; Arx, arabinoxylan; Gcm, glucomannan; Gtm, galactomannan; Glt, galactan; Xyl, xylan; Xlg, xyloglucan; Ac, amorphous cellulose; Lam, laminarin; Crd, curdlan; Pch, pachyman; Blg, Barley glucan; nd, test is not possible; potential new genera are in bold. *This isolate was similar to AArc-m2/3/4 isolated on mannan from soda lakes (see Table 2).

All isolates selected on alpha-bonded polysaccharides belonged to the well-characterized genera of haloarchaea, for some of which utilization of starch is known. However, to our knowledge, the capacity to utilize arabinan and arabinoxylan has never been shown/tested for any cultivated species of haloarchaea. This is also true for *Halorhabdus tiamateia*, a broadly-specialized polysaccharidolytic haloarchaeon, according to our current results (Table 1) and previous studies (Wainø and Ingvorsen, 2003; Werner et al., 2014). However, its ability to grow on levan (beta-fructan) was not known before. Isolates belonging to the genus *Natronoarchaeum* were second after *Halorhabdu*s by the number of isolated strains and were selected with four polysaccharides, including pullulan, beta-galactan, galactomannan, and curdlan. While utilization of galactomannan has already been reported for *N. mannanilyticum* (Shimane et al., 2010), growth with beta-1,4-galactan and beta-1,3-glucans, such as curdlan, have never been observed in any pure cultures of haloarchaea. Same is true for other two beta-1,4-bonded polysaccharides, including xyloglucan and beta-mannan. Looking from the taxonomical perspective, only two insoluble polysaccharides, mannan (consisted of beta-1,4-linked mannose) and curdlan (consisted of beta-1,3-linked glucose) (Table 1) resulted in selection of new genus-level isolates from the neutral salt lakes.

**FIGURE 1:**
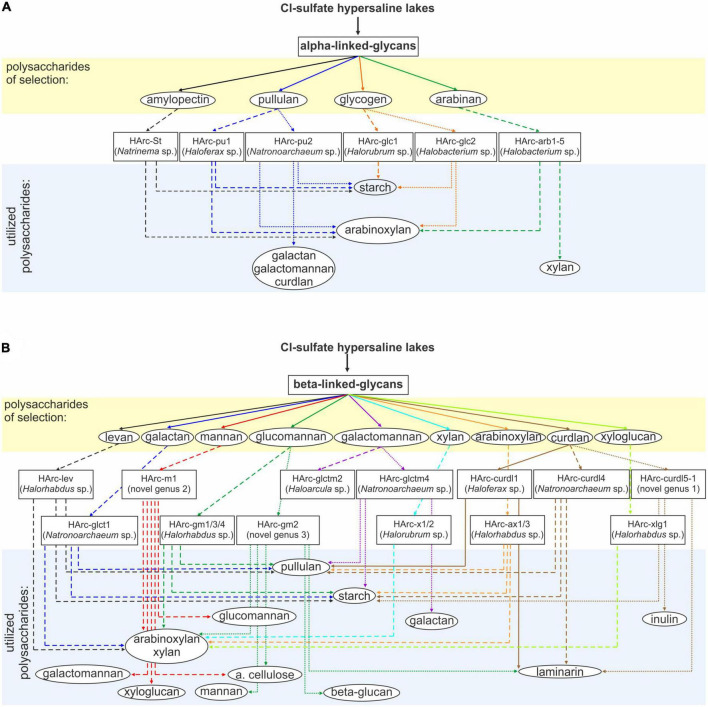
Schematic representation of selective enrichments of haloarchaea from hypersaline salt lakes on **(A)** alpha-bonded and **(B)** beta-bonded polysaccharides. Assignment to the novel genus was based on protein sequence-based phylogenomic analysis and 16S rRNA gene sequence identity values.

The cross-specificity for various polysaccharides supporting growth of the neutrophilic haloarchaea selected with a single polymer is shown in Figure 1. All five species enriched on alpha-glucans utilized soluble starch and five out of the six alpha-glucan specialists also grew with arabinoxylan which has alpha-bonded arabinose residues in the side chains. In turn, a single case of cross-specificity was also observed in *Halobacterium* strains which were selected with both glycogen (HArc-glc2) and arabinan (HArc-arb1-5) (Figure 1A).

Among the haloarchaeal strains selected with various beta-bonded polysaccharides the most common cross-substrates were xylan and arabinoxylan, while alpha-glucans (starch and pullulan) were only utilized by a few beta-glucan specialists (Figure 1B). Two out of the three isolates enriched with either mannan (HArc-m1) or glucomannan (HArc-gm2) were able to grow with amorphous cellulose indicating related selectivity of these beta-1,4 backbone polysaccharides. The *Halomicrobium* strain HArc-gm2 selected with glucomannan was identical in its 16S rRNA gene sequence to *Halomicrobium* sp. HArcel3–the cellulose-enriched haloarchaeon most closely related to *H. zhouii* (Sorokin et al., 2015). We tested the type strain *H. zhouii* JCM 17095 and it appeared to be able to grow with amorphous cellulose as well.

Strain HArc-m1 was identical (according to the 16S rRNA gene sequence analysis) to several natronoarchaeal isolates enriched from soda lakes with beta-1,4 mannan backbone polysaccharides (see below) confirming a link between the cellulose and the beta-mannan selectivity. This pattern has already been observed in cellulotrophic *Natronobiforma cellulositropha* which was enriched on cellulose but can also grow with beta-mannan (Sorokin et al., 2018). Furthermore, HArc-m1 is the only strain enriched from the neutral salt lakes being closely related to isolates from soda lakes representing quite a rare example in our long-term work with hypersaline lakes. It is also worth to notice that, in this particular case, the substrate selectivity (mannan) overruled the dominant selective factor – the nature of sodium salt (chloride vs. carbonate) and, therefore, the considerable difference in the pH-osmotic pressure combination. Growth experiments confirmed that strain HArc-m1 can indeed grow both at neutral pH and up to pH 9.5, thus being a facultative alkaliphile.

### Polysaccharide-utilizing natronoarchaea from hypersaline soda lakes

Positive stable enrichment cultures from soda lakes were obtained with 14 out of 16 polysaccharides tested (pullulan and arabinogalactan were not tested). Similar for salt lakes, alginate and pectin enrichments were negative. Another similarity was the fastest growth (1 week) of the starch-like alpha-glucans (amylopectin and glycogen) and the slowest growth (up to a month) of the insoluble beta-linked-glycans (beta-mannan and curdlan) and α-1,6-glucan dextran (which was negative in case of salt lake samples) utilizing enrichment cultures. However, in contrast to salt lakes further dilution to extinction from the soda lake enrichments had still to be done in the presence of antibiotics since in their absence the cultures were rapidly overrun by bacteria. It was even necessary to add antibiotics to the solid media at the final stage of pure culture isolation. The contaminating bacteria mostly belonged to the genus *Halomonas* which were unable to grow on the target polysaccharide but most probably scavenged the hydrolysis products. The *Halomonas* colonies were easily distinguished from the pink-orange colonies of natronoarchaea which helped to purify the latter, although, in most cases only 1-2 types of such colonies grew back in the liquid medium with the target polysaccharide. The list of isolated polysaccharide-utilizing natronoarchaea is given in Table 2.

**TABLE 2:** Polysaccharide-utilizing natronoarchaea enriched and isolated from hypersaline soda lakes.

Polysaccharide	Isolates	Identification by 16S rRNA gene sequence		Spectrum of utilized polysaccharides	
		Closest cultured relative	% identity	Stable growth in liquid culture	Colony activity (zone of hydrolysis, mm/2–3 weeks)
**Alpha-glucans**					
Amylopectin	AArc-St1-1 AArc-St1-2 AArc-St1-3 **AArc-St2**	*“Natranaeroarchaeum sulfidigenes”* *“Natronocalculus amylolyticus”*	99.4 98.5 99.1 **100**	Amp/Sst/Pul/Cdx/Lev Amp/Sst/Pul/Cdx/Inl	nd/35/23/nd/nd nd/19/15/nd/nd
Pullulan	Was not tested since AArc-St isolates were able to utilize pullulan				
Glycogen	AArc-glc1/2/4	*Natronococcus amylolyticus*	99	Glc/Sst/Arx/Xyl/Gcm	nd/23/25/15/20
	AArc-glc3	*Natronorubrum tibetense*	100	Glc/Sst	nd/25
Dextran	**AArc-dxtr1**	*Saliphagus infecundisoli*	96.8	Dxt/Arx/Sst(w)/Inl/Gcm(w)	nd/30/12/nd/8
Arabinan	AArc-arb1/2/6	*Natronolimnobius baerhuensis*	99	Arb/Arx/Arg/Xyl/Gcm(w)	nd/30/nd/9/11/5
	AArc-arb3/5	*Natrialba magadii*	96.4	Arb/Arx/Xyl	nd/15/25
**Beta-fructans**					
Levan	AArc-lev1	= AArc-St1-1	99	Lev/Sst	nd/20
Inulin	AArc-in1	= AArc-dxtr1	99.8	Inl/Sst/Dxt (weak)	nd/20/nd
	AArc-in2	=AArc-St2		Inl/Sst//Pul	nd/15/20
**Beta-bonded polysaccharides**					
Pectic galactan	AArc-glct1	*Natronolimnobius baerhuensis*	100	Glt/Arx/Xyl/Gcm/Arg	Nd/30/25/10/nd
Beta-mannan	AArc-m1	*Natronococcus amylolyticus*	99	Man/Arx/Xgl/Gcm	18/20/20/25
	AArc-m2/3/4*	*Halovarius/Haloterrigena*	**94–95**	Man/Gcm/Arx/Xyl/Xgl/Ac	20/20/30/30/20/20
	**AArc-m6**	*Natronobiforma cellulositropha*	100	Man/Arx/Xyl/Gcm/Ac	12/12/10/12/32
Glucomannan	**AArc-gm3/4/5-2**	**=AArc-m2/3/4**	100	Gcm/Man/Arx/Xyl/Cel	15/10/22/18/22
	**AArc-gm6**	*Natronobiforma cellulositropha*	100		
Galactomannan	**AArc-glctm3/4/8**	*Natronococcus amylolyticum*	99	Gtm/Gcm/Sst/Inl	nd/18/20/nd
	**AArc-glctm5**	=**AArc-m2/3/4**	**100**		
Xyloglucan	**AArc-xg1-1**	=**AArc-m2/3/4**	**100**	Xgl/Xyl/Arx/Ac/Man	15/7/22/12/8
Xylan arabinoxylan	AArc-x1/2/3/4	*Natronolimnobius baerhuensis*	99	Xyl/Arx/Ac/Sst	35/18/12/18
	AArc-ax1/2/3		99	Arx/Xyl/Arg	30/20/nd
Curdlan	**AArc-curdl1**	*Halostagnicola alkaliphila*	**95**	Crd/Pch/Lam/Gtm/Sst	nd/nd/nd/nd/15
Arabinogalactan	Was not tested since several other AArc isolates were able to utilize it				
Alginate	No isolates; not utilized by any isolates				
Pectin	There was some growth in primary enrichment but it was not reproduced further in sediment-free transfers				

See Table 1. w, weak growth; *This isolate was similar to HArc-m1 isolated on mannan from salt lakes (see Table 1). Bold values indicate potentially new genera.

Four different alpha-bonded glucans and fructans resulted in selection of seven isolates, of which, all except two belonged to know genera. Two isolates enriched and isolated either on amylopectin or inulin were identical in their 16S rRNA gene sequences and represented a novel genus and species *Natronocalculus amylovorans* (Sorokin et al., 2022a). The other three amylopectin-utilizing isolates were closely related to each other and to a facultively anaerobic sulfur-respiring amylolytic *Natranaeroarchaeum sulfidigenes* (Sorokin et al., 2022b). Together with the isolate AArc-lev selected with beta-fructan–levan (Table 2) those four strains have recently been described as a new species *Natranaeroarchaeum aerophilus* (Sorokin et al., 2022b). So, there seems to be a connection in starch-like alpha-glucans and beta-fructans selectivity among natronoarchaea.

Interestingly, although glycogen is structurally similar to amylopectin (both are branched alpha-glucans), the two glycogen-selected natronoarchaeal isolates from the genera *Natronococcus* and *Natronorubrum* were not related to above-mentioned new taxa. But all four were able to grow with soluble starch and pullulan, similar to the members of the genus *Natronococcus* which are well-known for their ability to utilize starch and to produce alkalistable amylases (Kobayashi et al., 1992; Kanal et al., 1995).

To our knowledge, dextrans have never been shown or even suspected to support growth of any known *Halobacteria* species, while it was mentioned among positive substrates in anaerobic hyperthermophilic archaea *Desulfurcococcus fermentas* (Perevalova et al., 2005), *D. kamchatkensis* (Kublanov et al., 2009), *Thermoccoccus sibiricus* (Mardanov et al., 2009), and *Thermococcus* sp. strain 2319 × 1 (Gavrilov et al., 2016). While starch-like polysaccharides can have side branches with alpha-bonded glucose other than α-1,4, none but dextrans have the α-1,6 backbone, which probably makes them difficult substrates for hydrolytic archaea. From two forms of the cyanobacterial dextran tested in this work (19.5 and 200 kDa), only the low molecular weight variety resulted in a positive enrichment and isolation of a single natronoarchaeal strain AArc-dxtr1 representing a distant novel species in the genus *Saliphagus* (Table 2).

Finally, an alpha-1,5-arabinan enrichment from soda lakes yielded a stable binary culture impossible to separate by serial dilutions. Plating showed two distinctive types of colonies: a dominant type with small red colonies and less abundant larger and nearly colorless colonies. Both grew back in liquid pure cultures with arabinan. Interestingly, the arabinan utilization in the liquid culture inoculated with the colorless colonies resulted in a formation of soluble yellow-brownish product, while the red colonies culture supernatant remained colorless. The isolate AArc-arb3/5 with colorless colonies was identified as a novel *Natrialba* species (with the highest 16S rRNA sequence identity of 97% to “*N. wudunaoensis*”), while the second isolate AArc-arb1/2/6 with red colonies was closely related to the known species *Natronolimnobius baerhuensis*.

The natronoarchaea selected from soda lakes on various beta-bonded polysaccharides can be divided into two major groups: preferably xylanolytic and cellulo-/mannanolytic (Table 2). The xylanolytics selected on either xylan, arabinoxylan and galactan belonged to the known genus *Natronolimnobius.* Xyloglucan, galactomannan, and mannan enrichments were all dominated by a novel genus-level lineage to which a facultatively alkaliphilic strain HArc-m1 (see above; Table 2) also belonged. Furthermore, a less abundant component in the galactomannan enrichment was identified as a member of the genus *Natronococcus.* A dominant organism in a glucomannan enrichment was identical to the cellulose/mannan-specialized *Natronobiforma cellulositropha* (Sorokin et al., 2018). This is similar to the selectivity of glucomannan in salt lakes resulted in isolation of a cellulolytic haloarchaeon HArc-gm2 closely related to cellulotrophic *Halomicrobium* HArcel3 dominating in cellulose enrichments from hypersaline lakes (Sorokin et al., 2015).

Curdlan, a beta-1,3-glucan homopolysacharide, selected a single natronoarchael strain representing a new genus lineage of the polysaccharide-utilizing archaea. The enrichment culture was very slow in development resulting in degradation of antibiotics and massive development of bacteria belonging to *Halomonas*. Several repeated attempts with sequential addition of antibiotics resulted in the sufficient enrichment of the archaeal component appropriate for further purification on a solid medium. Despite its general chemical similarity to cellulose, curdlan molecules have a different physical structure (helical in contrast to flat ribbon cellulose fibrils) (Deslandes et al., 1980). Altogether, its structural characteristics, both primary and secondary, as well as low occurrence in nature makes it highly selective substrate in comparison with the more common beta-1,4 glucans. We did not manage to find any published data on curdlan utilization in haloarchaea. On the other hand, two out of ten CAZymes families (GH81 and GH16) including members with the endo-β-1,3-glucanase activities (EC 3.2.1.39) have archaeal representatives. While archaeal representatives of the GH81 family are known exclusively by the presence of the respective genes in their genomes, a single archaeal GH16 glycosidase from *Pyrococcus furiosus* (Gueguen et al., 1997) was characterized as a laminarinase able to hydrolyze laminarin, lichenan, and barley β-glucan. However, no information of its capability to hydrolyze curdlan was provided. It should be noted that the halo- and natronoarchaea enriched and isolated on curdlan in the course of this work were capable to grow on laminarin, a soluble beta-1,3/1,6 glucan.

**FIGURE 2:**
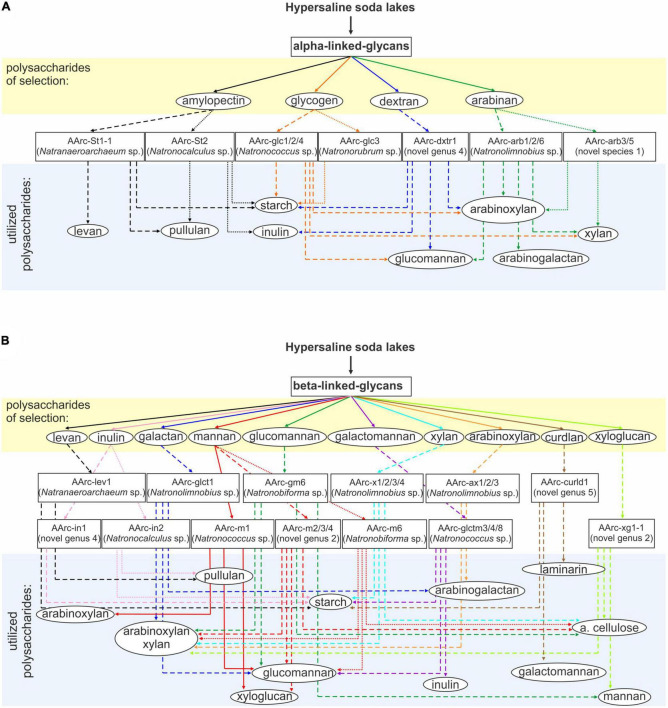
Schematic representation of selective enrichments of natronoarchaea from hypersaline soda lakes on **(A)** alpha-bonded and **(B)** beta-bonded polysaccharides. Assignment to the novel genus was based on protein sequence-based phylogenomic analysis and 16S rRNA gene sequence identity values (for strain AArc-arb3/5 only 16S rRNA gene sequence identity values were used).

The growth cross-specificity for various polysaccharides among the natronoarchaeal isolates is shown in Figure 2. From the strains isolated on alpha-bonded polysaccharides, the amylolytics were most restricted in their polymer-utilizing profiles with only beta-fructans as the alternative substrates. The only exception was *Natronococcus* AArc-glc1/2/4, isolated on glycogen and able to grow with a few beta-1,4 bonded polysaccharides. On the other hand, strains selected with other alpha-bonded polysaccharides, such as dextran and arabinan, were all able to utilize xylan and arabinoxylan and one of them also grew with arabinogalactan (Figure 2A).

Among the strains selected with beta-bonded polysaccharides, the most narrowly specialized was strain AArc-curdl1 isolated on curdlan: it only grew with two other polysaccharides with the beta-1,3-backbone (pachyman and laminarin) and on starch (Figure 2B). In contrast, the natronoarchaea enriched with various beta-1,4-bonded polysaccharides had a broader substrate range with xylan and arabonoxylan being the most common cross-substrates among them. Similar to the salt lake isolates, the soda lake strains selected with beta-1,4 mannan and glucomannan were also capable of growth on native celluloses. On the other hand, in contrast to salt lakes, the same taxa were also selected on galactomannan. This difference is significant, taking into account importance of cellulose for natural habitats but the reason for this is not clear yet.

### Phylogenomic and functional genomic analysis

Genomes of seven stains were *de novo* sequenced and assembled. Quality check of the assemblies revealed high completeness (99–100%) and low contamination (0-1.87%) levels what makes them suitable for both phylogenomic and functional analyses (Supplementary Table 2). Genome sizes varied from 2.81 to 5.59 Mbp while the G + C contents for different genomes were 59.2–66%.

**FIGURE 3:**
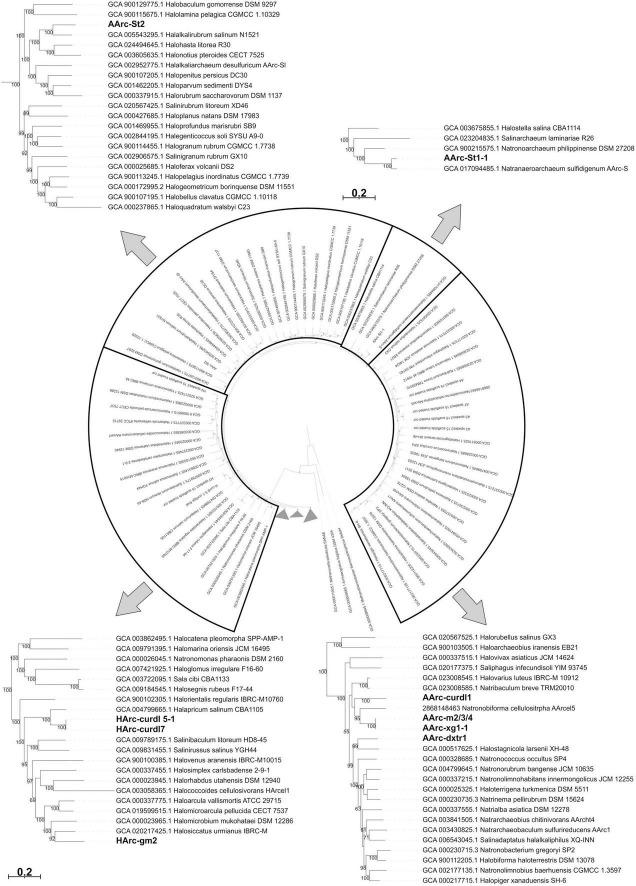
Maximum likelihood phylogenomic tree showing position of polysaccharide-utilizing halo(natrono)archaea enriched from hypersaline lakes within the class *Halobacteria*. Sequences of 122 conserved archaeal proteins were used to infer the tree.

Phylogenomic analysis showed that the novel polysaccharidolytic strains are uniformly dispersed within the *Halobacteria* tree (Figure 3). Strain AArc-St1-1 belonged to the genus *Natranaeroarchaeum* and is recently described as a new species *N. aerophilus* (Sorokin et al., 2022b), while strain AArc-St2 is described as a novel genus and species *Natronocalculus amylovorans* (Sorokin et al., 2022a). Strain HArc-gm2 was most closely related to the members of the genus *Halosiccatu*s. The neutrophilic curdlan-utilizing haloarchaea strains HArc-curdl5-1 and HArc-curdl7 are most closely related to the genus *Halapricum*. The nearest relatives of AArc-dxtr1 are among the *Halostagnicola* species, while AArc-xg1-1, AArc-m2/3/4 and AArc-cudrl1 are related to the cellulolytic *Natronobiforma*. Establishing the exact taxonomic rank (novel species or genus) of these novel haloa(natrono)archaea will need a more in-depth phenotypic and chemotaxonomical characterization.

**FIGURE 4:**
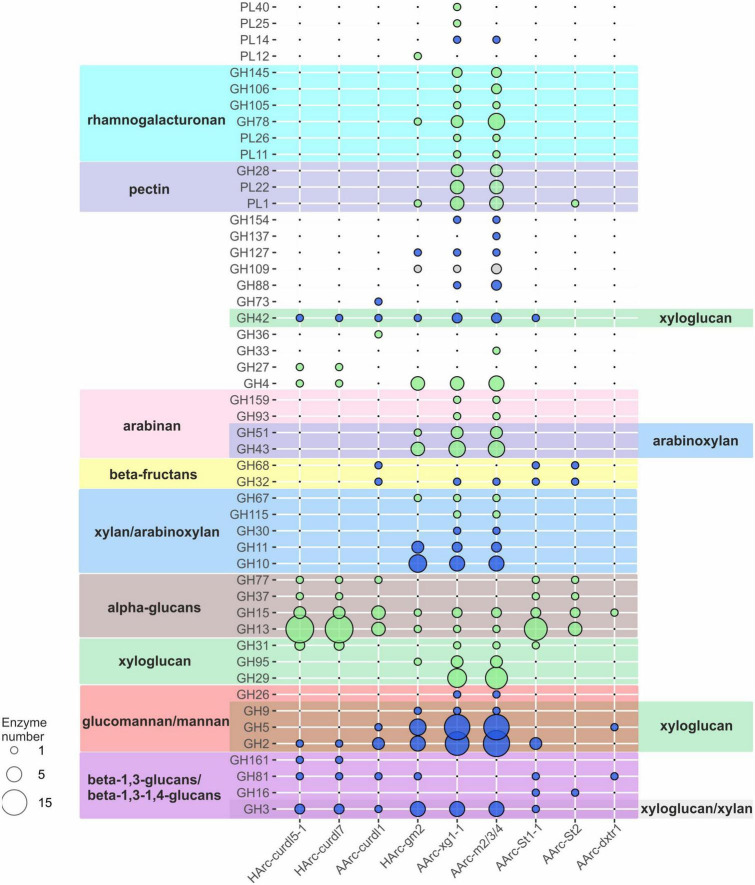
Sets of GH and PL genes found in the genomes of novel polysaccharidolytic haloarchaea. Size of bubbles indicate number of enzymes; the color of bubbles indicate the prevailing type of glycosidic bonds hydrolyzed by the enzymes (alpha-bonds—green, beta-bonds—blue, both—gray). Color of background indicates these CAZymes families contains the characterized enzyme(s) capable of depolymerization of respective polysaccharides. No color means the activities, characteristic to these families are not directly linked to the substrates used in this work.

Polysaccharide-utilizing haloarchaea must have a set of carbohydrate active enzymes (CAZymes), as a prerequisite for successful decomposition of insoluble and soluble poly- and oligosaccharides. Indeed, the sequenced genomes encoded all types of the CAZymes: glycosidases (GH), polysaccharide lyases (PL), glycosyl transferases (GT), carbohydrate esterases (CE), carbohydrate oxidases (AA) as well as carbohydrate-binding modules (CBM). The detailed analysis was focused on GHs and PLs (Figure 4; Supplementary Table 3) due to their major role in polysaccharide depolymerization.

Closely related neutrophilic strains HArc-curld5-1 and HArc-curdl7 enriched on curdlan had identical CAZyme repertoires. Their capability to degrade curdlan as well as pachyman and laminarin is due to the action of endo-beta-1,3(4)-glucanase (GH81), beta-1,3-glucan phosphorylase (GH161) and beta-glucosidase (GH3). Both strains also grew on starch by means of fourteen alpha-amylases (GH13), three oligo-1,6-glucosidases (GH13), two glucoamylases (GH15), and a 4-alphaglucanotransferase encoded in their genomes. The genome of alkaliphilic strain AArc-curld1 also isolated on curdlan had a smaller set of genes for respective enzymes yet it included an essential endo-beta-1,3(4)-glucanase (GH81) and a beta-glucosidase (GH3). A neopullulanase (GH13), three alpha-amylases (GH13), a 4-alphaglucanotransferase (GH77), and two glucoamylases (GH15) apparently allowed the strain to utilize soluble starch.

The neutrophilic strain HArcl-gm2 isolated on glucomannan has a machinery for its decomposition including endo-beta-1,4-mannosidase (GH5), several endoglucanases (four from the GH5 and one from the GH9 families), two beta-mannosidases (GH2) and a beta-glucosidase (GH3). The strain also can utilize laminarin and beta-glucan due to the presence of the endo-beta-1,3(4)-glucanase (GH81); xylan and arabinoxylan by means of endoxylanases (seven enzymes from GH10 and three from GH11), beta-xylosidases (GH3), and arabinosidases (GH43 and GH51) responsible for hydrolysis of side chains of arabinoxylan.

Comparative genomic analysis of closely related natronarchaeal strains AArc-xg1-1 (isolated on xyloglucan) and AArc-m2/3/4 (isolated on beta-1,4-mannan) showed a nearly identical and the largest CAZyme sets among the studied haloarchaea. In particular, the genes encoding several endo-beta-1,4-mannosidases (five enzymes from GH5 family and one from GH26), two beta-mannosidases (GH2), endoglucanases (10 proteins from GH5 and one enzyme from GH9), three beta-glucosidases (GH3 family), endo-beta-1,4-xylanases (5 enzymes from GH10 and two enzymes from GH11), beta-xylosidase (GH3) were found. Moreover, the genes of enzymes known to degrade galacturonate-containing polymers (polygalacturonate, pectin, rhamnogalacturonan) were also detected: three polygalacturonases (GH28), putative pectate lyases (four proteins from PL1 family and four from PL22), two rhamnogalacturonan lyases (PL11, PL26) as well as arabinan-hydrolyzing enzymes (six proteins from GH43, three GH51 enzymes and one enzyme from GH93). However, none of these isolates grew with either pectins, polygalacturonate, rhamnogalacturonan, or arabinan. This example clearly demonstrates that functional conclusions based solely on the genomic evidence should be considered only as preliminary.

The genome of the natronarchaeal amylolytic strain AArc-St1-1 (*Natranaeroarcheum aerophilus*, Sorokin et al., 2022a) contains a large number of genes coding for alpha-bond degrading glycosidases, including ten alpha-amylases (GH13 family), two oligo-1,6-glucosidases (GH13), 4-alpha-glucanotransferase (GH77), glucoamylase (GH15), and trehalase (GH37). Finally, two beta-fructosidases (GH32 and GH68) were also found, apparently allowing the strain to grow on levan. Also, two beta-1,3(4)-glucanases (GH16, GH81), beta-glucosidase/beta-xylosidase (GH3), beta-mannosidase (GH2), alpha-xylosidase (GH31), and several beta-galactosidases (GH2 and GH42 families) genes were present. Another amylolytic strain AArc-St2 (*Natronocalculus amylovorans*) possessed similar CAZymes set (alpha-glucan-specific enzymes from GH13, GH15 and GH77 families as well beta-fructosidases) but the number of alpha-amylases and oligo-1,6-glucosidases was significantly lower (Sorokin et al., 2020a). Despite the presence of genes for putative GH16 beta-glucanase and PL1 pectate lyase, no growth was observed on their specific substrates including beta-glucan, lichenan, curdlan, pachyman, or pectin.

Only three GH genes and no PL genes were found in the genome of dextran-utilizing natronoarchaeon AArc-dxtr1: trehalase (GH15), endoglucanase (GH5), and beta-1,3(4)-glucanase (GH81). The typical dextran-degrading enzymes from GH13, GH49, GH66, or GH70 families a were not encoded and it might be only speculated that dextran is hydrolyzed as a result of side activity of a GH15 enzyme since this family is known to contain glucodextranases (Mizuno et al., 2004). An endoglucanase and a beta-1,3(4)-glucanase could be responsible for hydrolysis of glucomannan and arabinoxylan which this organism can also utilize.

Altogether, the repertoire of CAZymes within the *in silico* proteomes of the nine polysacharidolytic halo/natronoarchaea analyzed in this work (summarized on Figure 4) was mostly consistent with their polysaccharide untilization spectrum. In total, the number of CAZyme genes found in the studied HArcel/AArcel strains greatly varied from 30 (strain AArc-St2) to 160 (strain AArc-m2/3/4). Closely related strains isolated with the same (HArc-curld5-1 and HArc-curdl7) or different (AArc-xg1-1 and AArc-m2/3/4) substrates may have identical or similar CAZymes gene sets indicating that a selection substrate will not necessarily lead to isolation of a distinctive phylogenetic lineage. At the same time, despite that the phylogenetically distant strains enriched and isolated with the same polysaccharide have different CAZymes sets, they all had similar patterns of particular CAZymes responsible for the hydrolysis of selective substrate. Surprisingly, the genomes of several isolates including AArc-xg1-1, AArc-m2/3/4, AArc-St2, and HArc-gm2 also contained polysaccharide lyases genes which are nearly unknown within the Archaeal kingdom. However, the growth experiments revealed that none of those strains can grow on alginate, pectin, polygalacturonate or rhamnogalacturonan indicating that these enzymes might have an unknown activity in halophilic archaea. This is also substantiated by the fact that all our attempts to enrich pectin- or alginate-utilizing haloarchaea failed so far. Among the possible reasons might be that these uronic acids-based polysaccharides are hardly present in hypersaline habitats or that hypersalinity changes the chemical properties and enzyme accessibility of the polymers. On the other hand, the high diversity of hydrolytic haloarchaea with the potential to utilize various polysacchirides with different types of glycosidic bonds indicate that such substrates might be available in hypersaline habitats. One of the most probable source of these polymers are external terrestrial plants growing in the area surrounding hypersaline lakes.

## Conclusion

The obtained results allow to significantly extend the knowledge on polysaccharide-utilizing capabilities of haloarchaea and archaea in general. Selective enrichment approach leaded to recover the so called “best-fit” organisms specialized on a narrow-specialized conversion of a particular substrate. In case of polysaccharides, however, most of the haloarchaeal isolates enriched with a certain substrate were still able to utilize several other polymers. It was also found that polymers with the alpha-1,4 or beta-1,4 linkage backbones more often resulted in positive enrichments than with other types of linkage, such as the alpha-1,6- or beta-1,3 bonding. Finally, no haloarchaea, growing on uronic acid-based polysaccharides (pectin and alginate), commonly utilized by bacteria, were isolated.

In the course of this work, the first haloarchaea able to grow on such recalcitrant polysaccharides as dextran, curdlan, xyloglucan, and beta-mannan were isolated. The enlarged variety of polysaccharidolytic halo(natrono)archaea recovered from hypersaline lakes with novel substrate utilization specificities is offering a good opportunity for further studies of their extremely halo(alkali)stable hydrolases, both in fundamental enzymology research and prospective application.

## Data availability statement

The datasets presented in this study can be found in online repositories. The names of the repository/repositories and accession number(s) can be found in the article/Supplementary material.

## Author contributions

DS and TVKh were responsible for microbiology work. AE and IK analyzed the genomes and run phylogenetic analysis. TVKo was responsible for 16S rRNA gene sequencing and identification of the isolates. DS, AE, and IK wrote the manuscript. All authors contributed to the article and approved the submitted version.
