# Supplementary material for: Selective enrichment on a wide polysaccharide spectrum allowed isolation of novel metabolic and taxonomic groups of haloarchaea from hypersaline lakes
Source: Front Microbiol. 2022 Nov 23;13:1059347. doi: 10.3389/fmicb.2022.1059347 (PMC9726719; doi:10.3389/fmicb.2022.1059347)
Supplement: Supplementary file 1 [file Data_Sheet_1.PDF]

Selective enrichment on a wide polysaccharide spectrum allowed isolation of novel metabolic and taxonomic groups of haloarchaea from hypersaline lakes.

Dimitry Y. Sorokin, Alexander G. Elcheninov, Tatjana Khizhniak, Tatjana V. Kolganova and Ilya V. Kublanov

Supplementary data file

Table S1. Polysaccharides used for selective enrichment of aerobic hydrolytic halo(natrono)archaea from hypersaline lakes.

Table S2. Statistics and quality of genome assemblies of isolated polysaccharidolytic haloarchaea.

Table S3. CAZymes genes found in the genomes of polysaccharidolytic halo(natrono)archaea.

Table S1. Polysaccharides used for selective enrichment of aerobic hydrolytic halo(natrono)archaea from hypersaline lakes

Polysaccharide	Source	Structure	Solubility in water
**alpha-glucans**			
amylopectin	Potato	α-1,4/1,6 glucan (branched)	insoluble
pullulan	*Aureobasidium pullulans*	α-1,4 glucan	soluble
glycogen	Oyster (clam)	α-1,4/1,6 glucan (branched)	partially soluble
dextran	*Leuconostoc*	α-1,6/1,3 glucan	soluble
arabinan	Sugar beat	α-1,5/1,3 polyarabinose	soluble
**beta-fructans**			
inulin	Tubers of *Chikory*	β-2,1 fructan	soluble
levan	Tymothy grass	β -2,6/2,1 fructan	soluble
**beta-glucans**			
pectic galactan	Potato	β-1,4 polygalactose	soluble
beta-mannan	Ivory nut	β-1,4 polymannose	insoluble (crystalline)
glucomannan	Konjak	β-1,4 mannan/glucan	insoluble (gel-forming)
galacto-mannan	Locust beans, (*Carob*)	**β-1,4 mannan - main chain** α-1,6 galactan - side chain	insoluble (gel-forming)
xylan	Birch and Beech woods	**β-1,4 polyxylose - main chain**	insoluble
xyloglucan	*Tamarind*	**β-1,4 glucan - main chain** α-1,6 xylane/ β-1,2 galactan/ α-1,2 arabinan - side chains	soluble
arabinoxylan	Rye	**β-1,4 xylan - main chain** α-1,3/1,2/1,5 arabinan - side chains	soluble
curdlan<br>pachyman	*Alcaligenes faecalis*<br>Fungal (*Poria*)	β-1,3 glucans	insoluble
arabinogalactan	Larch wood (*Larix liallii*)	**β-1,3 galactan - main chain** β-1,4/1,6 galactan/ α-1,6 arabinan - side chains	soluble
pectin	Citrus, apples	**β-1,4 galacturonate main chain**	insoluble
Na-alginate	Brown algae	β-1,4 guluronate/mannuronate	soluble

Supplemetary table S2. Statistics and quality of genome assemblies of isolated polysaccharidolytic haloarchaea

Strain	Genome size, Mbp	G+C, %	Number of contigs	N50, bp	Completeness, %	Contamination, %
AArc-dxtr1	2.81	63.3	5	1746851	100	0
AArc-curdl1	4.54	59.2	67	161529	100	1.4
AArc-xg1-1	5.59	62.1	67	201832	100	0.93
AArc-m2/3/4	5.51	62	46	203090	99.07	0.93
H-curdl5-1	3.36	63.1	62	116111	100	0.93
HArc-curdl7	3.38	63	52	159171	100	1.87
HArc-gm2	4.25	66	18	470222	100	0.93

Table S3: strain **AArc-St1-1**

**Locus tag**	**HMMER**	**Signal peptide (SignalP v6.0)**	**Function**
AArcSt11_00580	GT4(184-324)	-	GT
AArcSt11_01370	GH13(315-673)	Tat/SPI	alpha-amylase
AArcSt11_01380	GH13_32(67-338)	Tat/SPI	alpha-amylase
AArcSt11_03715	GH13_32(137-389)	Tat/SPII	alpha-amylase
AArcSt11_03720	GH13_20(293-645)	-	alpha-amylase
AArcSt11_04055	GT4(183-322)	-	GT
AArcSt11_04060	GT2_Glycos_transf_2(4-169)	-	GT
AArcSt11_05065	GH13_32(72-338)+CBM13(491-625)	Tat/SPI	alpha-amylase
AArcSt11_05620	GT2_Glycos_transf_2(6-134)	-	GT
AArcSt11_05715	GH13(270-561)	Tat/SPI	alpha-amylase
AArcSt11_07220	GH37(20-499)	-	trehalase
AArcSt11_07290	GH16(51-284)	Tat/SPII	beta-glucanase
AArcSt11_07650	GH15(287-667)	-	unknown
AArcSt11_07655	GH13(288-567)	-	alpha-amylase
AArcSt11_08270	GH13_20(306-629)	-	alpha-amylase
AArcSt11_08330	GH13(43-322)	no or Sec/SPI	alpha-amylase
AArcSt11_08825	GH15(287-650)+GH15(993-1388)	-	glucoamylase
AArcSt11_08830	GH77(13-487)	-	4-alpha-glucanotransferase
AArcSt11_09095	GT4(192-340)	-	GT
AArcSt11_09475	GH13(261-542)	-	alpha-amylase
AArcSt11_09505	GH13_31(35-378)	-	oligo-1,6-glucosidase
AArcSt11_09510	GH13_31(35-373)	-	oligo-1,6-glucosidase
AArcSt11_10040	GT66(34-675)	-	GT
AArcSt11_10245	GT2_Glycos_transf_2(9-188)	-	GT
AArcSt11_10395	GH31(169-598)	-	alpha-xylosidase
AArcSt11_10815	GH32(257-553)	-	beta-fructosidase
AArcSt11_10830	GH68(10-394)	-	beta-fructofuranosidase
AArcSt11_11000	GH81(77-713)	Tat/SPII	endo-1,3-beta-glucanase
AArcSt11_11010	GH3(90-311)	-	beta-xylosidase
AArcSt11_11550	GH2(562-966)	Tat/SPI	beta-galactosidase
AArcSt11_11555	GH2(11-816)	-	beta-galactosidase
AArcSt11_11560	GH42(6-385)	-	beta-galactosidase
AArcSt11_12085	GH2(20-555)	-	beta-mannosidase
AArcSt11_13975	GT2_Glycos_transf_2(4-150)	-	GT
AArcSt11_14230	GT81(99-289)	-	GT
AArcSt11_15975	GT4(185-321)	-	GT

Table S3: strain **AArc-St2**

**Locus tag**	**HMMER**	**Signal peptide** (SignalP v6.0)	**Function**
AArcSt2_00665	GT81(95-284)	-	GT
AArcSt2_00735	GT2_Glycos_transf_2(4-124)	-	GT
AArcSt2_00830	GT2_Glycos_transf_2(11-179)	-	GT
AArcSt2_01450	GH13_20(295-578)	-	alpha-amylase
AArcSt2_01455	GH15(289-664)	-	unknown
AArcSt2_04180	GH13_20(310-631)	Tat/SPI	alpha-amylase
AArcSt2_07575	GH32(257-554)	-	beta-fructosidase
AArcSt2_07580	GH68(24-404)	-	beta-fructofuranosidase
AArcSt2_07955	AA1(132-368)	Tat/SPII	multicopper oxidase MmcO
AArcSt2_08265	GH77(11-492)	-	4-alpha-glucanotransferase
AArcSt2_09525	GT4(179-315)	-	GT
AArcSt2_09610	AA7(38-407)	-	putative carbohydrate oxidase
AArcSt2_09745	GT81(20-200)	-	GT
AArcSt2_10530	GH13_31(35-392)	-	oligo-1,6-glucosidase
AArcSt2_10940	GT2_Glyco_tranf_2_3(5-221)	-	GT
AArcSt2_11025	GT4(197-341)	-	GT
AArcSt2_11040	GT4(188-339)	-	GT
AArcSt2_11045	GT4(195-343)	-	GT
AArcSt2_11300	GT2_Glycos_transf_2(4-168)	-	GT
AArcSt2_11305	GT66(41-730)	-	GT
AArcSt2_11675	GH37(27-499)	-	trehalase
AArcSt2_13715	GH16(26-261)	-	endo-1,3-1,4-beta-glucanase
AArcSt2_13720	GT2_Glyco_trans_2_3(103-297)	-	GT
AArcSt2_14250	GT2_Glycos_transf_2(9-182)	-	GT
AArcSt2_15130	AA7(36-407)	-	putative carbohydrate oxidase
AArcSt2_15190	PL1_2(117-291)	-	pectate lyase
AArcSt2_15275	GT4(211-360)	-	GT
AArcSt2_16150	GH13(285-569)	-	alpha-amylase
AArcSt2_16155	GH15(286-669)+GH15(1011-1402)	-	glucoamylase
AArcSt2_16605	GT4(182-322)	-	GT

Table S3: strain **AArc-dxtr1**

**Locus tag**	**HMMER**	**Signal peptide** (SignalP v6.0)	**Function**
OB905_00470	CBM57(258-374)+CBM57(461-571)	Sec/SPII	CBM
OB905_00495	CE14(8-117)	-	diacetylchitobiose deacetylase
OB905_00915	GH81(56-674)	Tat/SPI	endo-1,3(4)-beta-glucanase
OB905_01130	GH15(292-653)	-	trehalase
OB905_02070	CE14(8-117)	-	diacetylchitobiose deacetylase
OB905_02260	GT4(184-338)	-	GT
OB905_02685	GT2_Glycos_transf_2(9-191)	-	GT
OB905_02865	GT20(34-510)	-	GT
OB905_04265	CBM57(307-413)+CBM9(461-623)+CBM57(671-805)	Tat/SPII	CBM
OB905_04710	GT84(301-464)	Sec/SPII	GT
OB905_04715	CBM57(334-446)	Tat/SPII	CBM
OB905_04835	GT2_Glycos_transf_2(4-114)	-	GT
OB905_04890	GT81(105-288)	-	GT
OB905_05580	GT66(34-706)	-	GT
OB905_05585	GT2_Glycos_transf_2(4-168)	-	GT
OB905_05600	GT2_Glycos_transf_2(4-163)	-	GT
OB905_05605	GT2_Glycos_transf_2(16-178)	-	GT
OB905_05655	GT2_Glycos_transf_2(33-151)	-	GT
OB905_07190	GT4(226-380)	-	GT
OB905_07250	GH5(62-368)	Tat/SPI	endoglucanase
OB905_08350	GT2_Glyco_tranf_2_3(159-383)	-	GT
OB905_09375	CBM57(309-427)+CBM57(483-598)+CBM57(866-998)	Tat/SPII	CBM
OB905_10755	GT4(193-334)	-	GT
OB905_10845	GT4(163-311)	-	GT
OB905_10850	GT2_Glycos_transf_2(7-128)	-	GT
OB905_10865	GT4(207-364)	-	GT
OB905_10920	GT66(120-659)	-	GT
OB905_12615	CBM57(337-449)	Tat/SPII	CBM
OB905_12805	CBM57(339-442)	Tat/SPII	CBM
OB905_13355	CBM57(218-335)+CBM9(374-545)+CBM57(601-722)	Tat/SPII	CBM
OB905_13885	CBM57(320-442)+CBM57(457-602)	Tat/SPII	CBM

Table S3: AArc-curdl1

**Locus tag**	**HMMER**	**Signal peptide** (SignalP v6.0)	**Function**
OB919_00135	CBM34(8-130)+GH13_20(177-473)	-	neopullulanase
OB919_00155	GH15(317-728)	-	glucoamylase
OB919_00165	GH15(281-639)	-	unknown
OB919_00660	GT4(191-338)	-	GT
OB919_00740	AA7(36-408)	-	putative carbohydrate oxidase
OB919_00955	GH68(30-407)	-	beta-fructofuranosidase
OB919_01645	GT4(227-380)	-	GT
OB919_01700	GH2(3-594)	-	beta-mannosidase
OB919_02635	AA7(41-468)	-	putative carbohydrate oxidase
OB919_04360	CE14(4-113)	-	diacetylchitobiose deacetylase
OB919_04605	GH81(61-708)	Tat/SPI	endo-1,3(4)-beta-glucanase
OB919_04615	GH3(90-311)	-	beta-xylosidase
OB919_04770	GH2(15-502)	-	beta-galactosidase
OB919_04790	GH5_13(34-294)	-	unknown
OB919_04795	GH2(4-751)	-	beta-galactosidase
OB919_04935	AA7(34-458)	-	putative carbohydrate oxidase
OB919_05150	AA7(34-460)	-	putative carbohydrate oxidase
OB919_07805	GH13(353-689)+CBM13(856-996)	Tat/SPI	alpha-amylase
OB919_09760	CE1(46-225)	-	probable carboxylesterase
OB919_09840	GT81(107-296)	-	GT
OB919_10890	GT2_Glycos_transf_2(5-114)	-	GT
OB919_11965	GH13(238-521)	-	alpha-amylase
OB919_11970	GH15(277-653)+GH15(995-1386)	-	glucoamylase
OB919_11975	GH77(15-494)	-	4-alpha-glucanotransferase
OB919_12000	GH32(296-592)	-	beta-fructosidase
OB919_12295	GT4(186-327)	-	GT
OB919_12310	GT4(202-351)	-	GT
OB919_12920	GH15(235-593)	-	trehalase
OB919_13510	GH73(562-672)	Tat/SPI	putative murein hydrolase
OB919_13880	GT2_Glycos_transf_2(10-130)	-	GT
OB919_13890	GT2_Glycos_transf_2(6-170)	-	GT
OB919_13895	GT66(33-673)	-	GT
OB919_15140	CE4(224-348)	Sec/SPII or Tat/	unknown
OB919_17020	GT4(106-256)	-	GT
OB919_17040	CE4(60-190)	-	peptidoglycan deacetylase
OB919_17180	GT2_Glycos_transf_2(9-196)	-	GT
OB919_17185	CE4(48-162)	-	peptidoglycan deacetylase
OB919_18290	GT2_Glycos_transf_2(49-165)	-	GT
OB919_18550	GT20(41-529)	-	GT
OB919_19885	GH36(56-504)	-	alpha-galactosidase
OB919_19890	GH42(6-390)	-	beta-galactosidase
OB919_20390	GH13(305-655)	Tat/SPI	alpha-amylase
OB919_20490	AA7(33-404)	-	putative carbohydrate oxidase
OB919_20920	GT4(191-340)	-	GT

Table S3: strain **AArc-xg1-1**

Locus tag	HMMER	Signal peptide (SignalP v6.0)	Function
OB960_00040	GT4(184-320)	-	GT
OB960_00055	GT4(219-369)	-	GT
OB960_00060	GT2_Glycos_transf_2(4-168)	-	GT
OB960_00065	GT66(35-696)	-	GT
OB960_00460	GH15(248-609)	-	trehalase
OB960_01395	GT4(196-338)	-	GT
OB960_01410	GT4(202-350)	-	GT
OB960_02060	GH13_20(292-595)	-	alpha-amylase
OB960_02065	GH15(283-639)	-	glucoamylase
OB960_02575	GH105(40-342)	-	Unsaturated rhamnogalacturonyl hydrolase
OB960_02700	AA7(38-471)	-	putative carbohydrate oxidase
OB960_03335	GH3(52-260)	-	beta-glucosidase
OB960_04290	GH5(65-356)+CBM6(517-657)	Tat/SPI	endoglucanase
OB960_04295	GH5_7(92-386)	Tat/SPI	endo-1,4-beta-mannosidase
OB960_04300	GH5_7(96-387)	Tat/SPI	endo-1,4-beta-mannosidase
OB960_04315	GH5(66-371)	Tat/SPI	endoglucanase
OB960_04320	PL14_3(113-318)	Tat/SPI	unknown
OB960_04325	GH5(87-390)	Tat/SPI	endoglucanase
OB960_04340	GH5(97-403)	Tat/SPI	endoglucanase
OB960_04345	GH10(118-438)	Tat/SPI	celloxylanase/endo-1,4-beta-xylanase
OB960_04350	GH10(121-457)	Tat/SPI	endo-1,4-beta-xylanase
OB960_04355	GH10(117-443)	Tat/SPI	endo-1,4-beta-xylanase
OB960_04360	GH5(46-494)	Sec/SPI	endoglucanase
OB960_04365	GH5_7(108-396)	Tat/SPI	endo-1,4-beta-mannosidase
OB960_04375	GH5(68-392)	Tat/SPI	endoglucanase
OB960_04380	GH5(64-464)	Tat/SPI	endoglucanase
OB960_04385	GH5(84-402)	Tat/SPI	endoglucanase
OB960_04680	GT4(201-341)	-	GT
OB960_05170	GH3(90-314)	-	beta-glucosidase
OB960_05540	GH43_3(46-348)	Tat/SPI	exo-alpha-1,5-L-arabinofuranosidase
OB960_05580	AA7(34-458)	-	putative carbohydrate oxidase
OB960_06075	GH9(142-643)	Tat/SPI	endoglucanase
OB960_06245	GH43_12(5-284)	-	alpha-L-arabinofuranosidase
OB960_06735	PL1_2(110-300)	Tat/SPI	pectate lyase
OB960_06740	PL1_2(109-280)	Tat/SPI	pectate lyase
OB960_06875	GT87(71-307)	Sec/SPI or no	GT
OB960_06900	GH109(3-151)	-	D-glucoside 3-dehydrogenase
OB960_07565	GH145(28-333)	-	alpha-L-rhamnosidase
OB960_07570	GH42(6-387)	-	beta-galactosidase
OB960_07700	GH11(19-153)+CBM13(199-287	-	endo-1,4-beta-xylanase
OB960_07770	CBM85(41-175)	-	CBM
OB960_07965	GT20(10-480)	-	GT
OB960_08965	GH93(28-377)	-	exo-alpha-L-1,5-arabinanase
OB960_09825	GT2_Glycos_transf_2(5-114)	-	GT
OB960_10920	GH2(22-579)	-	beta-mannosidase
OB960_11235	GT2_Glycos_transf_2(9-175)	-	GT
OB960_11335	GT2_Glyco_tranf_2_3(87-303)	-	GT
OB960_11390	GH4(3-181)	-	alpha-galactosidase
OB960_12685	GH11(34-200)	Sec/SPI	endo-1,4-beta-xylanase
OB960_13415	PL11(1-588)	-	rhamnogalacturonan lyase
OB960_13425	GH43_18(45-275)	-	endo-1,5-alpha-L-arabinosidase
OB960_13430	GH95(7-795)	-	alpha-L-fucosidase
OB960_13460	PL22(44-149)+PL22(190-392)	-	oligogalacturonide lyase
OB960_13470	GH106(13-773)	-	unknown
OB960_13475	GH51(229-726)	-	alpha-L-arabinofuranosidase
OB960_13490	CBM67(119-302)+GH78(322-85	-	alpha-L-rhamnosidase
OB960_13505	PL22(44-149)+PL22_2(180-374)	-	oligogalacturonide lyase
OB960_13510	PL22_2(188-380)	-	oligogalacturonide lyase
OB960_13610	GH28(63-446)	-	polygalacturonase
OB960_13635	PL26(6-867)	-	rhamnogalacturonan exolyase
OB960_13640	GH2(5-701)	-	beta-glucuronidase
OB960_13650	GH2(60-833)	Tat/SPI	beta-galactosidase
OB960_13655	CBM13(565-705)	Tat/SPI	CBM
OB960_13815	PL1_2(529-716)	Tat/SPI	pectate lyase
OB960_13825	PL1_2(909-1102)	Tat/SPI	pectate lyase
OB960_13895	GH28(33-390)	-	polygalacturonase
OB960_14045	GH127(15-550)	-	beta-L-arabinofuranosidase
OB960_14065	GH2(9-665)	-	beta-glucuronidase
OB960_14095	GH43_3(32-328)	Tat/SPII	endo-alpha-1,5-L-arabinanase
OB960_14110	GH2(8-471)	-	beta-glucuronidase
OB960_14115	GH51(3-499)	-	alpha-L-arabinofuranosidase
OB960_14315	GH28(29-392)	-	polygalacturonase
OB960_14320	PL22(40-152)+PL22_2(203-381)	-	oligogalacturonide lyase
OB960_14325	CBM9(17-222)	-	CBM
OB960_14330	GH115(20-717)	-	alpha-1,2-glucuronidase
OB960_14340	GH4(20-200)	-	alpha-galacturonidase
OB960_14355	CBM67(358-531)+GH78(560-10	-	alpha-L-rhamnosidase
OB960_14360	CBM13(589-723)	Tat/SPI	CBM
OB960_14620	GH2(5-579)	-	beta-glucuronidase
OB960_14635	GH5(59-331)+CBM9(484-651)	Tat/SPII	endoglucanase
OB960_14745	GT4(180-276)	-	GT
OB960_14810	GT2_Glycos_transf_2(5-110)	-	GT
OB960_15085	GH2(36-945)	-	beta-galactosidase
OB960_15125	CE4(226-344)	Tat/SPII	polysaccharide deacetylase
OB960_15330	GT20(82-541)	-	GT
OB960_15510	GT2_Glyco_tranf_2_3(60-325)	-	GT
OB960_15565	CE4(3-135)	-	peptidoglycan deacetylase
OB960_15600	GT4(195-350)	-	GT
OB960_15620	GT2_Glycos_transf_2(5-113)	-	GT
OB960_15625	GT4(218-370)	-	GT
OB960_16100	GT2_Glycos_transf_2(18-156)	-	GT
OB960_16385	CE4(219-337)	Tat/SPII	putative polysaccharide deacetylase
OB960_16590	CE14(4-113)	-	diacetylchitobiose deacetylase
OB960_16665	CE14(17-126)	-	diacetylchitobiose deacetylase
OB960_16820	GH32(24-329)	-	beta-fructosidase
OB960_16825	GH154(11-359)	-	putative beta-glucuronidase
OB960_17195	GH31(221-716)	-	alpha-xylosidase
OB960_17200	GH5(67-371)	Tat/SPI	endoglucanase
OB960_17950	CE14(6-115)	-	diacetylchitobiose deacetylase
OB960_18050	CE15(84-403)+CE15(442-766)	-	4-O-methyl-glucuronoyl methylesterase
OB960_18060	PL40(597-926)	-	putative ulvan lyase
OB960_18145	CE14(9-118)	-	diacetylchitobiose deacetylase
OB960_18930	PL25(2-133)	-	ulvan lyase
OB960_19135	GT2_Glycos_transf_2(33-178)	-	GT
OB960_19520	GT4(204-342)	-	GT
OB960_19530	GT4(195-345)	-	GT
OB960_19545	GT4(181-331)	-	GT
OB960_19580	GT2_Glycos_transf_2(70-230)	-	GT
OB960_19595	CE15(19-396)	-	carbohydrate esterase
OB960_20140	GT4(226-382)	-	GT
OB960_20475	GH3(43-254)	-	beta-glucosidase
OB960_20885	GH43_12(5-279)	-	alpha-L-arabinofuranosidase
OB960_20890	GH159(23-239)	-	beta-D-galactofuranosidase
OB960_21020	GT2_Glyco_tranf_2_3(46-263)	-	GT
OB960_21120	GH4(3-181)	-	alpha-galactosidase
OB960_21555	GH2(41-943)	-	beta-galactosidase
OB960_21560	GH2(36-930)	-	beta-galactosidase
OB960_21575	GH145(28-311)	-	aplha-L-rhamnosidase
OB960_21580	GH30_4(135-587)	-	endo-beta-1,6-galactanase
OB960_21645	GH2(54-645)	Sec/SPII or Sec/	beta-glucuronidase
OB960_21710	GH88(44-379)	-	unsaturated glucuronyl hydrolase
OB960_21830	GH95(8-739)	-	alpha-L-fucosidase
OB960_21835	GH29(3-348)	-	alpha-L-fucosidase
OB960_21920	GH3(86-315)	-	beta-xylosidase
OB960_22090	GH2(3-711)	-	beta-mannosidase
OB960_22105	GH5_8(96-291)	Tat/SPI	endo-1,4-beta-mannosidase
OB960_22110	GH43_3(44-331)+CBM13(361-5	Tat/SPI	endo-alpha-1,5-L-arabinanase
OB960_22120	GH26(67-364)	Tat/SPI	endo-1,4-beta-mannosidase
OB960_22140	GH5_7(89-381)	Tat/SPII	endo-1,4-beta-mannosidase
OB960_23595	GH29(4-360)	-	alpha-L-fucosidase
OB960_23605	GH29(15-352)	-	alpha-L-fucosidase
OB960_23610	GH29(4-348)	-	alpha-L-fucosidase
OB960_23615	GH29(8-315)	-	alpha-L-fucosidase
OB960_24985	CBM67(325-482)+GH78(544-10	-	alpha-L-rhamnosidase
OB960_24990	GH2(6-568)	-	beta-glucuronidase
OB960_25000	GH2(2-507)	-	beta-galactosidase
OB960_25020	GH42(25-404)	-	beta-galactosidase
OB960_25025	CBM13(418-510)	Tat/SPI	CBM
OB960_25065	GH29(3-373)	-	alpha-L-fucosidase
OB960_25070	GH29(5-351)	-	alpha-L-fucosidase
OB960_25175	GH10(8-318)	-	endo-1,4-beta-xylanase
OB960_25180	GH4(29-206)	-	alpha-galacturonidase
OB960_25190	GH3(66-292)	-	beta-xylosidase
OB960_25225	GH67(8-688)	-	alpha-glucuronidase
OB960_25230	CBM85(106-238)+CBM85(329-4	Tat/SPI	endo-1,4-beta-xylanase
OB960_25235	CBM6(2-87)	-	CBM
OB960_25580	GH29(3-299)	-	alpha-L-fucosidase
OB960_25600	GH95(7-734)	-	alpha-L-fucosidase
OB960_25715	GH51(289-807)	Tat/SPII	alpha-1,5-L-arabinofuranosidase

Table S3: strain **AArc-m2/3/4**

**Locus tag**	**HMMER**	**Signal peptide** (SignalP v6.0)	**Function**
OB955_01610	GH3(52-260)	-	beta-glucosidase
OB955_02250	AA7(38-471)	-	putative carbohydrate oxidase
OB955_02375	GH105(40-342)	-	unsaturated rhamnogalacturonyl hydrolase
OB955_02890	GH15(283-639)	-	unknown
OB955_02895	GH13_20(292-598)	-	alpha-amylase
OB955_03545	GT4(202-350)	-	GT
OB955_03560	GT4(196-338)	-	GT
OB955_04535	GH11(34-200)	Sec/SPI	endo-1,4-beta-xylanase
OB955_05955	GH5(67-371)	Tat/SPI	endoglucanase
OB955_05960	GH31(221-716)	-	alpha-xylosidase
OB955_06040	GH11(76-245)+CBM13(291-380)+CBM13(358-426)	Tat/SPI	endo-1,4-beta-xylanase
OB955_06045	CBM6(2-87)	-	CBM
OB955_06050	CBM85(106-238)+CBM85(329-460)+GH10(518-835)	Tat/SPI	endo-1,4-beta-xylanase
OB955_06055	GH67(8-688)	-	alpha-1,2-glucuronosidase
OB955_06090	GH3(66-292)	-	beta-xylosidase
OB955_06100	GH4(29-206)	-	alpha-galacturonidase
OB955_06105	GH10(8-318)	-	endo-1,4-beta-xylanase
OB955_06205	GH42(6-387)	-	beta-galactosidase
OB955_06210	GH145(28-333)	-	aplha-L-rhamnosidase
OB955_06740	GH109(3-151)	-	D-glucoside 3-dehydrogenase
OB955_06765	GT87(71-307)	Sec/SPI	GT
OB955_06995	AA7(34-458)	-	putative carbohydrate oxidase
OB955_07035	GH43_3(46-348)	Tat/SPI	endo-alpha-1,5-L-arabinanase
OB955_07405	GH3(90-314)	-	beta-glucosidase
OB955_07895	GT4(201-341)	-	GT
OB955_08185	GH5(84-402)	Tat/SPI	endoglucanase
OB955_08190	GH5(64-464)	Tat/SPI	endoglucanase
OB955_08195	GH5(68-392)	Tat/SPI	endoglucanase
OB955_08205	GH5_7(108-396)	Tat/SPI	endo-1,4-beta-mannosidase
OB955_08210	GH5(46-494)	Sec/SPI	endoglucanase
OB955_08215	GH10(117-443)	Tat/SPI	endo-1,4-beta-xylanase
OB955_08220	GH10(121-461)	Tat/SPI	endo-1,4-beta-xylanase
OB955_08225	GH10(118-438)	Tat/SPI	celloxylanase/endo-1,4-beta-xylanase
OB955_08230	GH5(97-403)	Tat/SPI	endoglucanase
OB955_08245	GH5(87-390)	Tat/SPI	endoglucanase
OB955_08250	PL14_3(113-318)	Tat/SPI	unknown
OB955_08255	GH5(66-371)	Tat/SPI	endoglucanase
OB955_08270	GH5_7(64-354)	Sec/SPI	endo-1,4-beta-mannosidase
OB955_08275	GH5_7(92-386)	Tat/SPI	endo-1,4-beta-mannosidase
OB955_08280	GH5(65-356)+CBM6(517-657)	Tat/SPI	endoglucanase
OB955_09465	GH109(3-365)	-	alpha-N-acetylgalactosaminidase
OB955_09620	GT2_Glycos_transf_2(33-178)	-	GT
OB955_09650	GT2_Glycos_transf_2(4-166)	-	GT
OB955_09655	GT2_Glycos_transf_2(4-168)	-	GT
OB955_09660	GT66(35-696)	-	GT
OB955_10060	GH15(248-609)	-	trehalase
OB955_10930	GH93(28-377)	-	alpha-L-arabinofuranobiosidase
OB955_11765	GH51(3-499)	-	alpha-L-arabinofuranosidase
OB955_11770	GH2(8-471)	-	beta-glucuronidase
OB955_11785	GH43_3(32-328)	Tat/SPII	endo-alpha-1,5-L-arabinanase
OB955_11815	GH2(9-667)	-	beta-glucuronidase
OB955_11835	GH127(15-550)	-	beta-L-arabinofuranosidase
OB955_11990	GH28(33-390)	-	polygalacturonase
OB955_12060	PL1_2(1108-1301)	Tat/SPI	pectate lyase
OB955_12070	PL1_2(529-716)	Tat/SPI	pectate lyase
OB955_12230	CBM13(565-705)	Tat/SPI	CBM
OB955_12235	GH2(60-833)	Tat/SPI	beta-galactosidase
OB955_12245	GH2(5-482)	-	beta-glucuronidase
OB955_12250	PL26(6-867)	-	rhamnogalacturonan exolyase
OB955_12275	GH28(63-446)	-	polygalacturonase
OB955_12375	PL22_2(188-380)	-	oligogalacturonate lyase
OB955_12380	PL22(44-149)+PL22_2(180-374)	-	oligogalacturonide lyase
OB955_12395	CBM67(119-302)+GH78(322-852)	-	alpha-L-rhamnosidase
OB955_12410	GH51(230-726)	-	alpha-L-arabinofuranosidase
OB955_12415	GH106(13-765)	-	putative alpha-L-rhamnosidase
OB955_12425	PL22(46-151)+PL22(192-394)	-	oligogalacturonide lyase
OB955_12455	GH95(7-795)	-	alpha-L-fucosidase
OB955_12460	GH43_18(45-275)	-	putative endo-1,5-alpha-L-arabinosidase
OB955_12470	PL11(1-588)	-	rhamnogalacturonan lyase
OB955_12485	CBM67(325-482)+GH78(544-1065)	-	alpha-L-rhamnosidase
OB955_12490	GH2(6-568)	-	beta-glucuronidase
OB955_12500	GH2(2-507)	-	beta-galactosidase
OB955_12520	GH42(25-404)	-	beta-galactosidase
OB955_12525	CBM13(418-510)+CBM13(487-557)	Tat/SPI	CBM
OB955_12630	PL1_2(109-280)	Tat/SPI	pectate lyase
OB955_12635	PL1_2(110-300)	Tat/SPI	pectate lyase
OB955_13125	GH43_12(5-284)	-	alpha-L-arabinofuranosidase
OB955_13295	GH9(142-643)	Tat/SPI	endoglucanase
OB955_13755	CE14(4-113)	-	diacetylchitobiose deacetylase
OB955_13830	CE14(17-126)	-	diacetylchitobiose deacetylase
OB955_13985	GH32(24-329)	-	beta-fructosidase
OB955_13990	GH154(11-359)	-	putative beta-glucuronidase
OB955_15065	GH2(41-943)	-	beta-galactosidase
OB955_15075	GH2(36-930)	-	beta-galactosidase
OB955_15090	GH145(28-311)	-	alpha-L-rhamnosidase
OB955_15095	GH30_4(135-587)	-	endo-beta-1,6-galactanase
OB955_15160	GH2(54-645)	Sec/SPII or Sec/S	beta-glucuronidase
OB955_15220	GH88(44-378)	-	unsaturated glucuronyl hydrolase
OB955_15340	GH95(8-739)	-	alpha-L-fucosidase
OB955_15345	GH29(3-348)	-	alpha-L-fucosidase
OB955_15370	GH2(5-579)	-	beta-galactosidase
OB955_15385	GH5(59-331)+CBM9(484-651)	Tat/SPII	endoglucanase
OB955_15495	GT4(180-276)	-	GT
OB955_15565	GT2_Glycos_transf_2(5-109)	-	GT
OB955_15825	GH2(36-945)	-	beta-galactosidase
OB955_15865	CE4(226-344)	Tat/SPII	putative polysaccharide deacetylase
OB955_16070	GT20(87-541)	-	GT
OB955_16185	GH3(43-254)	-	beta-glucosidase
OB955_16595	GH43_12(5-279)	-	alpha-L-arabinofuranosidase
OB955_16600	GH159(23-239)	-	putative beta-D-galactofuranosidase
OB955_16830	GH4(3-181)	-	alpha-galactosidase
OB955_17315	GT2_Glycos_transf_2(5-115)	-	GT
OB955_18455	CE4(219-337)	Tat/SPII	putative polysaccharide deacetylase
OB955_18740	GT2_Glycos_transf_2(18-156)	-	GT
OB955_19385	GT4(204-342)	-	GT
OB955_19395	GT4(195-345)	-	GT
OB955_19405	GT4(179-331)	-	GT
OB955_19435	GT2_Glycos_transf_2(62-222)	-	GT
OB955_19450	CE15(19-396)	-	carbohydrate esterase
OB955_19975	GT8(1-224)	-	GT
OB955_19980	GT2_Glycos_transf_2(9-154)	-	GT
OB955_19985	GT4(202-343)	-	GT
OB955_20065	GT4(200-341)	-	GT
OB955_20370	GH4(3-181)	-	alpha-galactosidase
OB955_20515	GT2_Glycos_transf_2(9-175)	-	GT
OB955_20830	GH2(22-579)	-	beta-mannosidase
OB955_21395	GH51(290-806)	Tat/SPII	alpha-1,5-L-arabinofuranosidase
OB955_21595	GH28(29-392)	-	polygalacturonase
OB955_21600	PL22(40-152)+PL22_2(203-381)	-	oligogalacturonide lyase
OB955_21605	CBM9(17-222)	-	CBM
OB955_21610	GH115(21-728)	-	alpha-glucuronidase
OB955_21620	GH4(20-200)	-	alpha-galacturonidase
OB955_21635	CBM67(359-507)+GH78(567-1080)	-	alpha-L-rhamnosidase
OB955_21640	CBM13(589-723)	Tat/SPI	CBM
OB955_21990	GT2_Glycos_transf_2(141-292)	-	GT
OB955_21995	GT2_Glycos_transf_2(52-178)	-	GT
OB955_22025	GT2_Glycos_transf_2(22-144)	-	GT
OB955_22425	GH88(40-374)	-	unsaturated glucuronyl hydrolase
OB955_22455	GH29(2-352)	-	alpha-L-fucosidase
OB955_22465	GH137(47-345)	-	putative beta-L-arabinofuranosidase
OB955_22485	GH2(29-582)+GH2(808-1117)	-	beta-galactosidase
OB955_22490	GH2(30-696)	-	beta-galactosidase
OB955_22525	GH29(3-339)	-	alpha-L-fucosidase
OB955_22530	GH2(31-818)	-	beta-galactosidase
OB955_22540	GH29(13-380)	-	alpha-L-fucosidase
OB955_22590	CBM67(114-298)+GH78(320-840)	-	alpha-L-rhamnosidase
OB955_22595	GH106(12-746)	-	alpha-L rhamnosidase
OB955_22610	CBM67(339-504)+GH78(530-1037)	-	alpha-L-rhamnosidase
OB955_22620	GH33(23-339)	-	putative sialidase
OB955_22640	CBM67(141-307)+GH78(331-838)	-	alpha-L-rhamnosidase
OB955_22770	GH5_7(89-381)	Tat/SPII	endo-1,4-beta-mannosidase
OB955_22790	GH26(67-364)	Tat/SPI	endo-1,4-beta-mannosidase
OB955_22795	GH43_3(44-331)+CBM13(361-502)	Tat/SPI	endo-alpha-1,5-L-arabinanase
OB955_22800	GH5_8(96-291)	Tat/SPI	endo-1,4-beta-mannosidase
OB955_22815	GH2(3-691)	-	beta-mannosidase
OB955_22985	GH3(86-315)	-	beta-xylosidase
OB955_23495	GT20(10-480)	-	GT
OB955_23685	CBM85(71-205)	-	CBM
OB955_24005	GT2_Glyco_tranf_2_3(87-303)	-	GT
OB955_24060	GH4(3-181)	-	alpha-galactosidase
OB955_24200	GH95(7-734)	-	alpha-L-fucosidase
OB955_24390	GT4(225-382)	-	GT
OB955_25125	GH29(8-315)	-	alpha-L-fucosidase
OB955_25130	GH29(4-348)	-	alpha-L-fucosidase
OB955_25135	GH29(15-352)	-	alpha-L-fucosidase
OB955_25145	GH29(4-360)	-	alpha-L-fucosidase
OB955_25560	GH29(5-351)	-	alpha-L-fucosidase
OB955_25565	GH29(3-174)	-	alpha-L-fucosidase
OB955_25600	GH29(15-209)	-	alpha-L-fucosidase

Table S3: strain **HArc-curdl5-1**

**Locus tag**	**HMMER**	**Signal peptide** (SignalP v6.0)	**Function**
OB916_00345	GH15(314-667)	-	glucoamylase
OB916_00405	GH13_20(277-566)	-	alpha-amylase
OB916_00500	GH13(281-595)	-	alpha-amylase
OB916_01050	GH2(17-600)	-	exo-beta-D-glucosaminidase
OB916_01685	GH13(285-575)	-	alpha-amylase
OB916_01715	GH13_31(28-393)	-	oligo-1,6-glucosidase
OB916_02035	GH13(305-685)	Tat/SPI	alpha-amylase
OB916_02345	GT4(186-297)	-	GT
OB916_02400	GT75(87-370)	-	GT
OB916_02430	GT2_Glycos_transf_2(10-165)	-	GT
OB916_02715	GH13_4(114-512)+CBM20(663-734)	-	amylosucrase
OB916_02720	GH13_16(30-385)	-	trehalose synthase/amylase
OB916_02730	GH13_31(28-371)	-	oligo-1,6-glucosidase
OB916_02740	GH13_31(39-384)	-	oligo-1,6-glucosidase
OB916_03150	GH81(63-741)+CBM6(1032-1168)	Tat/SPI	endo-1,3(4)-beta-glucanase
OB916_03200	GH3(95-321)	-	beta-xylosidase
OB916_03355	GT81(6-185)	-	GT
OB916_03680	GH15(300-664)	-	unknown
OB916_03740	GH77(11-490)	-	4-alpha-glucanotransferase
OB916_03760	GH13(253-531)	-	alpha-amylase
OB916_03765	GT35(173-517)	-	glycogen phosphorylase
OB916_04015	GH13_16(30-383)	-	trehalose synthase/amylase
OB916_04285	GH13_20(292-570)	Tat/SPI	alpha-amylase
OB916_04550	GH13(280-595)	-	alpha-amylase
OB916_05655	GT81(99-284)	-	GT
OB916_06405	GT2_Glycos_transf_2(36-148)	-	GT
OB916_06415	CE4(31-142)	-	peptidoglycan deacetylase
OB916_06515	GH13(17-327)	-	alpha-amylase
OB916_07025	GH13(321-613)	Tat/SPII	alpha-amylase
OB916_07685	GT2_Glycos_transf_2(5-122)	-	GT
OB916_08130	GT4(178-321)	-	GT
OB916_08575	GH15(321-671)	-	glucoamylase
OB916_08850	GT66(123-614)	-	GT
OB916_08935	GT66(32-522)	-	GT
OB916_09025	GH31(172-599)	-	alpha-xylosidase
OB916_09030	GH31(170-597)	-	alpha-xylosidase
OB916_09050	GT2_Glycos_transf_2(39-200)	-	GT
OB916_09055	GT4(213-346)	-	GT
OB916_09065	GT4(202-335)	-	GT
OB916_09090	GT4(199-342)	-	GT
OB916_09325	GT2_Glycos_transf_2(8-174)	-	GT
OB916_10135	GT87(72-225)	-	GT
OB916_10280	GT66(31-651)	-	GT
OB916_10285	GT2_Glycos_transf_2(5-173)	-	GT
OB916_10290	GT4(204-325)	-	GT
OB916_10305	GT4(204-312)	-	GT
OB916_10350	GH13(59-326)	-	alpha-amylase
OB916_10585	GT2_Glyco_trans_2_3(173-363)	-	GT
OB916_10690	GH42(6-387)	-	beta-galactosidase
OB916_10715	GH27(109-368)	-	alpha-galactosidase
OB916_10720	GH4(4-181)	-	alpha-galactosidase
OB916_10745	GH13_20(295-633)	-	alpha-amylase
OB916_11155	GT2_Glycos_transf_2(9-192)	-	GT
OB916_12495	GT35(171-488)	-	glycogen phosphorylase
OB916_12725	GH13_32(48-280)	-	alpha-amylase
OB916_13250	GH37(27-505)	-	trehalase
OB916_14480	AA7(41-340)	-	putative carbohydrate oxidase
OB916_15020	GT4(191-313)	-	GT
OB916_15420	GT4(182-323)	-	GT
OB916_15425	GT4(200-338)	-	GT
OB916_15735	GH3(40-248)	-	beta-glucosidase
OB916_16085	GT83(139-434)	-	GT
OB916_16280	GT4(151-301)	-	GT
OB916_16390	GT2_Glycos_transf_2(11-170)	-	GT
OB916_16405	GT4(187-327)	-	GT
OB916_16440	GT4(191-324)	-	GT
OB916_16495	AA7(35-461)	-	putative carbohydrate oxidase
OB916_16585	GH161(1-1060)	-	beta-1,3-glucan phosphorylase

Table S3: strain **HArc-curdl7**

**Locus tag**	**HMMER**	**Signal peptide** (SignalP v6.0)	**Function**
OB914_00280	GH13_31(39-384)	-	oligo-1,6-glucosidase
OB914_00290	GH13_31(28-371)	-	oligo-1,6-glucosidase
OB914_00300	GH13_16(30-385)	-	trehalose synthase/amylase
OB914_00305	GH13_4(114-512)+CBM20(663-734)	-	amylosucrase
OB914_00590	GT2_Glycos_transf_2(10-165)	-	GT
OB914_00620	GT75(87-370)	-	GT
OB914_00675	GT4(186-297)	-	GT
OB914_00985	GH13(305-685)	Tat/SPI	alpha-amylase
OB914_01305	GH13_31(28-393)	-	oligo-1,6-glucosidase
OB914_01335	GH13(285-575)	-	alpha-amylase
OB914_01970	GH2(17-600)	-	exo-beta-D-glucosaminidase
OB914_02520	GH13(281-595)	-	alpha-amylase
OB914_02615	GH13_20(277-566)	-	alpha-amylase
OB914_02675	GH15(314-667)	-	glucoamylase
OB914_03085	GH161(1-1060)	-	beta-1,3-glucan phosphorylase
OB914_03225	GH81(55-733)+CBM6(1024-1160)	Tat/SPI	endo-1,3(4)-beta-glucanase
OB914_03275	GH3(95-321)	-	beta-xylosidase
OB914_03430	GT81(6-185)	-	GT
OB914_03755	GH15(300-664)	-	unknown
OB914_03815	GH77(11-490)	-	4-alpha-glucanotransferase
OB914_03835	GH13(253-531)	-	alpha-amylase
OB914_03840	GT35(173-517)	-	glycogen phosphorylase
OB914_04090	GH13_16(30-383)	-	trehalose synthase/amylase
OB914_04360	GH13_20(292-570)	Tat/SPI	alpha-amylase
OB914_04625	GH13(280-595)	-	alpha-amylase
OB914_05085	GT4(199-342)	-	GT
OB914_05110	GT4(202-335)	-	GT
OB914_05120	GT4(213-346)	-	GT
OB914_05125	GT2_Glycos_transf_2(39-200)	-	GT
OB914_05145	GH31(170-597)	-	alpha-xylosidase
OB914_05150	GH31(172-599)	-	alpha-xylosidase
OB914_05240	GT66(32-522)	-	GT
OB914_05445	GT66(123-614)	-	GT
OB914_05585	GT4(204-312)	-	GT
OB914_05600	GT4(204-325)	-	GT
OB914_05605	GT2_Glycos_transf_2(5-173)	-	GT
OB914_05610	GT66(31-651)	-	GT
OB914_05755	GT87(72-225)	-	GT
OB914_06990	GT81(99-284)	-	GT
OB914_07210	GH13(17-327)	-	alpha-amylase
OB914_07310	CE4(31-142)	-	peptidoglycan deacetylase
OB914_07320	GT2_Glycos_transf_2(36-148)	-	GT
OB914_08360	GH13(321-613)	Tat/SPII	alpha-amylase
OB914_09025	GT2_Glycos_transf_2(5-122)	-	GT
OB914_09475	GT4(6-149)	-	GT
OB914_09955	GT2_Glycos_transf_2(8-174)	-	GT
OB914_10385	GH15(321-671)	-	glucoamylase
OB914_10740	GH13_20(295-633)	-	alpha-amylase
OB914_10765	GH4(4-181)	-	alpha-galactosidase
OB914_10770	GH27(109-368)	-	alpha-galactosidase
OB914_10795	GH42(6-387)	-	beta-galactosidase
OB914_10900	GT2_Glyco_trans_2_3(173-363)	-	GT
OB914_11135	GH13(72-339)	-	alpha-amylase
OB914_11200	GT2_Glycos_transf_2(9-192)	-	GT
OB914_12880	GT35(171-488)	-	glycogen phosphorylase
OB914_13365	GH37(27-505)	-	trehalase
OB914_13620	GH13_32(48-280)	-	alpha-amylase
OB914_14180	GT4(151-301)	-	GT
OB914_15255	AA7(41-340)	-	putative carbohydrate oxidase
OB914_15565	GT4(191-313)	-	GT
OB914_15955	GT4(182-323)	-	GT
OB914_15960	GT4(200-338)	-	GT
OB914_16065	GH3(53-261)	-	beta-glucosidase
OB914_16185	GT83(139-434)	-	GT
OB914_16330	GT4(191-324)	-	GT
OB914_16365	GT4(187-327)	-	GT
OB914_16380	GT2_Glycos_transf_2(11-170)	-	GT
OB914_16575	AA7(35-461)	-	putative carbohydrate oxidase

Table S3: strain **HArc-gm2**

**Locus tag**	**HMMER**	**Signal peptide** (SignalP v6.0)	**Function**
OB920_01505	GT4(181-321)	-	GT
OB920_01510	GT4(198-337)	-	GT
OB920_01535	GH3(40-247)	-	beta-glucosidase
OB920_03705	GT81(4-188)	-	GT
OB920_03720	GT4(206-339)	-	GT
OB920_03755	GT4(208-354)	-	GT
OB920_03760	GT2_Glycos_transf_2(7-121)	-	GT
OB920_03765	GT66(35-658)	-	GT
OB920_05495	GH3(80-307)	-	beta-xylosidase
OB920_06225	GH5(69-384)	Tat/SPI	endoglucanase
OB920_06230	GH5(63-434)	Tat/SPI	endoglucanase
OB920_06240	CE6(131-235)+CBM6(362-498)	Tat/SPI	carbohydrate acetyl esterase/feruloyl esterase
OB920_06250	GH5(82-366)+CBM6(521-656)	Tat/SPI	endoglucanase
OB920_06255	GH10(108-434)	Tat/SPI	endo-1,4-beta-xylanase
OB920_06260	GH5(84-385)	Tat/SPI	endoglucanase
OB920_06590	GH10(12-319)	-	endo-1,4-beta-xylanase
OB920_06600	GH4(19-195)	-	alpha-galacturonidase
OB920_06605	CBM85(93-228)+GH10(294-596)	Tat/SPI	endo-1,4-beta-xylanase
OB920_07170	GH3(89-316)	-	beta glucosidase
OB920_07405	GH2(19-368)	-	beta-mannosidase
OB920_07500	CBM85(91-226)+GH10(287-588)	Tat/SPI	endo-1,4-beta-xylanase
OB920_08310	GT4(194-342)	-	GT
OB920_08320	GT4(215-375)	-	GT
OB920_08325	GT2_Glycos_transf_2(5-162)	-	GT
OB920_08345	GT4(190-350)	-	GT
OB920_08395	GT66(112-597)	-	GT
OB920_08980	CBM6(72-171)	-	CBM
OB920_09185	GH4(3-181)	-	alpha-galactosidase
OB920_09195	GH4(3-180)	-	alpha-galactosidase
OB920_09210	GH2(29-934)	-	beta-galactosidase
OB920_09290	GH43_3(45-346)+CBM13(399-535)	Tat/SPI	endo-alpha-1,5-L-arabinanase
OB920_09435	GT2_Glycos_transf_2(9-162)	-	GT
OB920_09470	GT2_Glycos_transf_2(5-114)	-	GT
OB920_09545	GT75(5-374)	-	GT
OB920_09570	GT2_Glycos_transf_2(14-164)	-	GT
OB920_09800	GH9(157-612)	Tat/SPII	endoglucanase
OB920_09985	GH109(1-154)	-	D-glucoside 3-dehydrogenase
OB920_10135	PL1_2(99-283)+CBM13(477-615)	Tat/SPI	pectate lyase
OB920_10880	GT66(23-652)	-	GT
OB920_10920	CE4(5-119)	-	chitooligosaccharide deacetylase
OB920_11685	GT2_Glycos_transf_2(4-109)	-	GT
OB920_11805	GT2_Glycos_transf_2(7-175)	-	GT
OB920_11850	GT81(108-293)	-	GT
OB920_13550	GH3(94-319)	-	beta-xylosidase
OB920_13785	CBM13(107-261)	Sec/SPI	CBM
OB920_13885	GH10(91-403)	Tat/SPI	endo-1,4-beta-xylanase
OB920_14610	GT20(25-496)	-	GT
OB920_14830	GT2_Glycos_transf_2(9-185)	-	GT
OB920_14850	CBM35(408-521)	Tat/SPI	CBM
OB920_15560	GH2(9-557)	-	beta-galactosidase
OB920_15950	GH5_8(81-278)	Tat/SPI	endo-1,4-beta-mannosidase
OB920_16560	CE15(38-429)	-	carbohydrate esterase
OB920_16960	GT2_Glycos_transf_2(7-111)	-	GT
OB920_16965	GT4(198-343)	-	GT
OB920_16970	GT4(207-353)	-	GT
OB920_16980	PL12(374-512)	-	heparin-sulfate lyase
OB920_16985	GT4(220-374)	-	GT
OB920_17000	GT2_Glycos_transf_2(146-304)	-	GT
OB920_17400	GT2_Glycos_transf_2(61-169)	-	GT
OB920_17495	CE4(115-223)	Tat/SPII	chitooligosaccharide deacetylase
OB920_17560	GH81(82-708)+CBM56(801-940)	Tat/SPI	endo-1,3(4)-beta-glucanase
OB920_17715	GH2(3-785)	-	beta-mannosidase
OB920_18365	GT4(165-298)	-	GT
OB920_18845	CE1(25-275)	-	esterase
OB920_18985	GH95(8-740)	-	alpha-L-fucosidase
OB920_18995	GH43_12(5-279)	-	alpha-L-arabinofuranosidase
OB920_19135	GH42(6-398)	-	beta-galactosidase
OB920_19540	GH15(291-652)	-	unknown
OB920_19545	GH13_20(289-575)	-	alpha-amylase
OB920_19595	GT87(69-311)	-	GT
OB920_19985	CBM13(3-90)	-	CBM
OB920_19990	GH11(77-250)+CBM13(288-423)	Tat/SPI	endo-1,4-beta-xylanase
OB920_19995	GH10(109-407)+CBM6(512-648)	Tat/SPI	endo-1,4-beta-xylanase
OB920_20000	GH11(39-213)	Tat/SPI	endo-1,4-beta-xylanase
OB920_20005	GH11(75-249)+CBM6(303-439)	Tat/SPI	endo-1,4-beta-xylanase
OB920_20010	CBM85(75-210)+GH10(281-584)	Sec/SPI	endo-1,4-beta-xylanase
OB920_20015	GH67(8-688)	-	alpha-1,2-glucuronosidase
OB920_20075	GH43_12(4-279)	-	alpha-L-arabinofuranosidase
OB920_20130	GH2(7-659)	-	beta-galactosidase
OB920_20140	GH51(3-495)	-	alpha-L-arabinofuranosidase
OB920_20170	GH127(17-560)	-	beta-L-arabinofuranosidase
OB920_20255	CBM67(342-509)+GH78(536-1041)	-	alpha-L-rhamnosidase
OB920_20260	GH4(17-195)	-	alpha-galacturonidase
OB920_20275	CBM13(454-592)	Tat/SPI	CBM
OB920_20280	GH43_3(45-332)+CBM13(411-547)	Tat/SPI	endo-alpha-1,5-L-arabinanase
OB920_20300	GH3(93-316)	-	beta-xylosidase
